# Supplementary material for: A complete telomere-to-telomere assembly of the maize genome
Source: Nat Genet. 2023 Jun 15;55(7):1221–31. doi: 10.1038/s41588-023-01419-6 (PMC10335936; doi:10.1038/s41588-023-01419-6)
Supplement: Supplementary file 1 — Supplementary Text, Supplementary Methods, Supplementary Note, Supplementary Figs. 1–17, Supplementary Tables 1–14 and Supplementary References. [file 41588_2023_1419_MOESM1_ESM.pdf]

nature genetics

Article https://doi.org/10.1038/s41588-023-01419-6

A complete telomere-to-telomere assembly of the maize genome

In the format provided by the authors and unedited

Supplementary Text

Supplementary Methods

Supplementary Notes

Supplementary Figures 1-17

Supplementary Tables 1-14

References

Supplementary Text

Integration of the basal Mo17 assembly with a PacBio assembly

By utilizing ultra-long ONT reads, we obtained the basal Mo17 assembly, including 10 pseudomolecules with only 10 gaps, with each of chr3, chr4, chr5, chr7, and chr10 covered by one single contig (**Fig. 1b**). To systematically validate the basal Mo17 assembly and find potential assembly errors, quality filtered ultra-long ONT reads > 10 kb were mapped to the assembly. In addition to existing gaps and terminal regions of chromosomes, there were only 12 low coverage regions (LCRs) with read depth lower than 100 and 10 high coverage regions (HCRs) with read depth higher than 250 (**Fig. 1b**), which were mainly related to super-long microsatellites, satellites and rDNAs arrays, as well as highly repetitive TE regions. The coverage of remained genomic regions were relatively uniform, with an average of 177×.

We then assembled the Mo17 genome based on 69.4× PacBio HiFi data. Both the assemblers Hifiasm[1] and Canu[2] were used, resulting in assemblies of 2.71 Gb (contig N50 of 51.4 Mb) and 2.27 Gb (contig N50 of 39.9 Mb), respectively (**Supplementary Table 2**). According to the alignment, the PacBio contigs which can span the gaps or correct the assembly errors of the ONT-based assembly were integrated into the basal Mo17 assembly. Five gaps in the basal Mo17 assembly were closed by PacBio contigs, including three transposable element (TE) related gaps on chr2, chr6 and chr9, a 1.7 Mb CentC repeat array related gap, and a 15.1 Mb knob180 repeat array related gap on chr8 (**Supplementary Fig. 2**). For the 12 LCRs, 6 included assembly errors, including LCR6 on chr4 which was split forming a new gap (termed as gap_LCR6) (**Supplementary Fig. 3**), and 5 LCRs which errors were corrected by the PacBio assembly (**Extended Data Fig. 2**). The assemblies of these gap-closed and corrected regions were further confirmed by the uniform ONT-read coverage and tiling of ONT reads (**Fig. 1c and 1d**, **Extended Data Fig. 2 and 3**). One LCR corrected by the PacBio assembly was a 556.4 kb rDNA array on chr2, which harbored a total of 1,387 copies of 5S rDNAs. A blast-based copy number estimation (see method) was performed using both ultra-long ONT data and PacBio HiFi data, which showed there were 1,319 and 1,312 copies of 5S rDNAs in the genome, respectively (**Fig. 2a**). The Illumina data was used for the estimation using a k-mer-based method (see method), and showed there were 1,128 5S rDNA copies in the genome (**Fig. 2a**). The numbers

of 5S rDNAs estimated were highly similar to those assembled for the 5S rDNA array on chr2, the only genomic region with 5S rDNA.

The remaining 6 LCRs and 10 HCRs, which assemblies were all confirmed by concordant PacBio assembly and tiling ONT reads, were due to local sequence features and not assembly error (**Supplementary Fig. 4 and 5**). Low coverage of 4 LCRs related to TAG repeat arrays longer than 9 kb, 2 LCRs related to satellite arrays longer than 100 kb (one was Cent4 reported previously[3], and one was a new satellite discovered here) was introduced by mapping errors plus extra miscalled errors for ONT reads with long TAG repeats (**Supplementary Fig. 6**). Notably, in the final Mo17 assembly, the sequence errors of the 4 TAG repeat array related LCRs introduced by ONT reads were corrected by corresponding PacBio contigs or ONT reads in which TAG repeats were not miscalled as other microsatellites (**Supplementary Fig. 4**). For the 10 HCRs, 5 were related to genomic regions with homolog sequences at the mitochondrial genome, 4 were related to TEs, and one was related to subtelomeric repeat array longer than 150 kb (**Supplementary Fig. 5 and 7**), which high coverage was introduced by mapping errors of reads from their corresponding homolog sequences. With the ONT reads longer than 50 kb, the coverage of 9 HCRs was relatively uniform, except for HCR8 with 2 copies of super-long tandem repeat unit (about 300 kb) (**Supplementary Fig. 5)**.

Compared to the Mo17ref_V1[4], there was a large inversion around 96-103 Mb on chr4 (**Extended Data Fig. 1**). The basal Mo17 assembly in this region, which was validated by the concordant PacBio assembly and uniform ONT-read coverage (**Supplementary Fig. 8**), was consistent with the assemblies all 25 Nested Association Mapping (NAM) founder lines and B73[5]. This suggested earlier anchoring and orientation errors for contigs of the Mo17ref_V1 assembly.

Finishing the 20 telomeric ends

Chromosomal ends were often depleted in read coverage. Here, we checked the assembly of all 20 terminal 1 Mb regions of chromosomes. No structural error was found between ONT and PacBio assemblies (**Supplementary Fig. 9**). Notably, and similar to TAG repeats, telomeric repeats (5’-TTTAGGG-3’) could be missed due to

sequencing errors in the ONT reads. As indicated by the presence of over 2 kb of telomeric repeats in the assembly, 18 chromosomal ends were fully assembled by both ONT and PacBio approaches. Incomplete chromosomal ends were found on the short arm of chr1 (chr1S) and chr2S (**Supplementary Fig. 9**). The telomeric region of chr1S was assembled by ONT data only. While for the end of chr2S, the telomeric region was found in only the PacBio Canu assembly, and therefore, the corresponding PacBio contig was integrated into the basal Mo17 assembly (**Supplementary Fig. 9**). To avoid telomeric repeat sequences being incorrectly trimmed by the assembler, we further corrected telomeric regions using ONT reads. Thus, we obtained the assemblies of the ends of all 10 chromosomes, as confirmed by tiling ONT reads and coverage analysis (**Fig. 1E**, **Extended Data Fig. 4**).

Gap closure of 5 super-long TAG trinucleotide repeat arrays

Following gap-closing and correction by the PacBio assembly, there were only 6 gaps remaining in the basal Mo17 assembly, including 5 related to super-long TAG repeat arrays on chr1 (gap1 and gap2), chr2 (gap3 and gap5) and chr4 (gap_LCR6) and one related to the 45S rDNA array on chr6 (gap6). These six highly repetitive gaps were too long (all longer than 200 kb) to be spanned by current ultra-long ONT reads directly. According to previous reports[6,7], we found there were three types of reads with sequencing errors of ONT technology after carefully examining reads falling into these gaps (**Supplementary Fig. 6**): (1) Symmetrical read: reads generated due to a given DNA fragment being mistakenly sequenced twice from opposite directions. (2) Fused read: reads containing sequences apparently fused from two or more genomic regions. (3) Microsatellites (also known as simple sequence repeats) miscalled reads: a stretch of microsatellites in reads were miscalled as other microsatellites, including many reads with long TAG repeats or telomeric repeats. Two or more types of these errors could happen in a single read. With a base error rate of approximately 15%[8,9], these additional sequencing errors of current ONT technology were a major reason that gaps were not easily filled using a typical genome assembler.

Specific gap-filling approaches were developed for gaps related to super-long TAG repeats and 45 rDNAs, respectively. We tried to close the 5 super-long TAG repeat array related gaps by manual extension using the ultra-long ONT reads. Gap1 and

gap5 were spanned by 6 and 16 tiling ONT reads, respectively (**Fig. 1f, Extended Data Fig. 5**). The assembly showed that the TAG repeat array related to gap1 was 375 kb, among which 67.64% of sequences were TAG repeats (**Supplementary Table 3**). Gap5 was much longer (1.56 Mb), including 890.9 kb of TAG repeat sequences (**Supplementary Table 3**). Gap2, gap3 and gap_LCR6 were also extended for about 700 kb, 100 kb, and 1 Mb, respectively. However, each of these still included one TAG repeat region that was not spanned by ONT reads, which were termed sub-gap2, sub-gap3, and sub-gap_LCR6, respectively. Further analyses indicated that TAG repeat sequences harbored in sub-gap2, sub-gap3, and sub-gap_LCR6 were all longer than 90 kb (**Extended Data Fig. 5**). We hypothesized that these remaining gaps might contain only TAG trinucleotide repeats, allowing few point mutations. Based on our earlier analyses that the TAG repeats could often be base-called as other microsatellites, we manually checked 4,129 reads with at least 5 kb of microsatellites at one end of the read (out of all 77× quality-passed ONT reads longer than 100 kb). With the exception of 504 reads composed only of microsatellite repeats, the remaining 3,625 reads were mapped back to other parts of our Mo17 genome assembly, confirming that sub-gap2, sub-gap3, and sub-gap_LCR6 all contain only TAG repeat sequences. We then tried to determine their length using BioNano physical molecules of the Mo17 genome[4]. The length of sub-gap_LCR6 was approximately 129 kb according to one BioNano molecule that spanned it (**Extended Data Fig. 5**). Sub-gap3 could be spanned by a total of 7 reliable BioNano molecules. Interestingly, they varied in their estimated length of sub-gap3, with length differences ranging up to 70 kb (**Supplementary Fig. 10**). These findings are consistent with previous reports showing the length of highly similar tandem repeats can vary greatly among individuals due to unequal crossover mediated expansions and contractions of repeat arrays[10]. We estimated the length of sub-gap3 to be 210 kb, the median of the 7 BioNano molecules (**Extended Data Fig. 5, Supplementary Fig. 10**). Next, we tried to determine the length of sub-gap2. Briefly, total length of all 6 genomic regions with consecutive TAG repeats longer than 90 kb was estimated first using 45.6× ONT reads longer than 150 kb, including one region in each of gap1, gap3, gap_LCR6, and two regions in gap5, as well as sub-gap2. The estimated total length of the 6 genomic regions (1,025 kb) was then subtracted by the lengths of the 5 regions with known sizes. Thus, the length of sub-gap2 was determined to be approximately 166 kb. In

summary, gap2, gap3, and gap_LCR6 were closed, with lengths of 637.39 kb, 211.26 kb, and 1.13 Mb, respectively (**Extended Data Fig. 5**).

To validate the accuracy of our assembly for these 5 super-long TAG repeat array related gaps, we performed a fluorescence in situ hybridization (FISH) assay using pachytene stage meiocytes. A total of 6 identifiable TAG repeat signals were identified using such sequences as probes. According to their relative positions on chromosomes, these signals were found to correspond to the longest 6 TAG repeat arrays in the genome, including those in the 5 TAG repeat array related gaps and that in a TAG repeat array related LCR (LCR3) (**Fig. 2b**). In addition, the intensities of these FISH signals were generally consistent with the lengths of TAG repeats inferred from our assembly (**Fig. 2b**), thus confirming the accuracy of the assembly of these super-long TAG repeat arrays.

Gap closure of 45S rDNA array

We closed the 45S rDNA related gap using PacBio HiFi reads. The intergenic spacer region (IGS) sequence of 45S rDNA was utilized as the main anchor for closure as there was abundant genetic variation[11-13]. First, two internal 'islands' with opposite directions of 45S rDNAs were identified by PacBio HiFi and ONT reads. As we decided to extend along the transcriptional direction of 45S rDNAs, three locations served as starting points of extension, including two sides of an 'island' with rDNAs in which IGSs were adjacent to each other, and the centromere-proximal end of the gap in which the 45S rDNAs direction was toward the telomere (**Extended Data Fig. 6**). During the extension steps, PacBio HiFi reads were utilized because of their high base accuracy in order to distinguish genetic variation among different 45S rDNA copies. In addition, the N50 size of PacBio HiFi reads was about 15.9 kb, nearly two-fold longer than the 45S rDNA repeat unit, which made it possible to extend one copy of 45S rDNAs most of the time. Briefly, extensions were made using an in-house script as follows: the IGS sequence of the 45S rDNA to be extended was mapped to the PacBio reads harboring 45S rDNAs, and the 5 best hit reads were selected and mapped back to the corresponding 45S rDNA to be extended. The read with the best hit was further selected for extension. In some cases, manual checks and extension were needed when TE insertions occurred or no PacBio reads were found

based on the thresholds set for this best-hit-based method. Overall, the 45S rDNA related gap was closed by PacBio reads with a total of 3,106 rounds of extension, with a total length of 26.8 Mb containing 2,974 copies of 45S rDNAs. The 1.2 Mb TE-enriched region in the centromere-proximal end of the array was the only region which could also be reliably assembled by ONT reads. The number and the order of 45S rDNAs in this TE-enriched region were highly consistent across assemblies based on PacBio and ONT reads (**Fig. 1g**). To further validate the accuracy of the assembly of the array, the number of 45S rDNA copies was then estimated by different approaches. The blast-based copy number estimation with ultra-long ONT and PacBio HiFi data showed that there were 2,662 and 2,849 copies of 45S rDNAs in the Mo17 genome, respectively (**Fig. 2c**). The k-mer-based estimation with Illumina data indicated that the number of 45S rDNAs in the Mo17 genome was 2,694 (**Fig. 2c**). In addition, digital PCR based experimental estimations were also performed, demonstrating there were 3,364 ± 237 copies of 45S rDNAs (**Fig. 2c**). Overall, the total 45S rDNA copies estimated were highly consistent with the 45S rDNA array assembly, the only genomic region having 45S rDNAs in Mo17 genome.

Evaluation of the accuracy of the final T2T Mo17 assembly using ONT and PacBio reads

We remapped the ultra-long ONT reads and PacBio HiFi reads to the T2T Mo17 assembly and found uniform coverage across nearly all genomic regions, which confirmed the overall accuracy of the assembly (**Extended Data Fig. 7**). According to the alignments of ONT reads, except for the 20 telomeres that often show underrepresentation of reads and one subtelomeric region on chr2S with highly tandem repeats (**Extended Data Fig. 4**), we observed 15 LCRs and 10 HCRs with read depth lower than 100 and higher than 250 (genome-wide average: 180.7), respectively, which all corresponded to gaps, LCRs and HCRs mentioned for the basal Mo17 assembly (**Supplementary Fig. 11**). Based on PacBio HiFi reads, 107 additional local coverage-anomalous regions with coverage lower than 20 or higher then 105 (genome-wide average: 65) were identified. Notably, the assemblies of these regions, with an average size of 20.84 kb, were all confirmed by concordant PacBio assembly and tiling ONT reads.

Evaluation of the completeness of the final T2T Mo17 assembly using ONT reads

The completeness of the T2T assembly was validated. Of 7,751,268 quality-passed ONT reads longer than 10 kb, 0.47% originated from maize mitochondrion and chloroplast genomes and microbial DNA contamination as identified by mapping with organelle genomes and the NCBI NT database, and 6.21% were generated due to sequencing errors, including 3.60% fused reads and 1.54% symmetrical reads, as well as 1.07% reads of unknown mistaken origin. The remaining 93.32% reads could be properly mapped to a unique position of the T2T Mo17 assembly with a minimum query sequence coverage of 0.85 (**Extended Data Fig. 8**). We did not detect quality-passed ONT reads that originated from the Mo17 genome but failed to map to the final assembly.

Analysis of tandem repeats in the Mo17 genome

With a requirement of at least 5 consecutive copies, we identified 5.45 Mb of microsatellites (repeat unit: 1 to 9 nucleotides), 16.43 Mb of minisatellites (repeat unit: 10 to 100 nucleotides), 36.98 Mb of satellites (repeat unit: > 100 nucleotides), 0.44 Mb of 5S rDNAs, and 26.08 Mb of 45S rDNAs, which collectively accounted for 3.92% of the Mo17 genome (**Supplementary Table 4**). Notably, about 12.57% of microsatellites, 98.88% of minisatellites, and 7.90% of satellites were identified in TE regions. Compared to the Mo17ref_V1[4], about 3.4 Mb microsatellites (73.4% were in 5 super-long TAG repeat arrays), 33.3 Mb satellites (68.4% were in two knobs at chr6S and 8L, and 19.6% were in centromeric regions), and almost all (99.8%) rDNAs were newly assembled here. Totally, this complete assembly added (~85%) or corrected (~15%) 127.15 Mb of sequence that did not linearly aligned to the pseudomolecules of the Mo17ref_V1[4] with Mummer[14], including 4.41 Mb of non-repetitive sequences (**Supplementary Table 4**).

Genes annotated in the Mo17 genome

A total of 42,580 high-confidence, protein-coding genes were annotated in the Mo17 genome, of which 30,975 were supported by RNA-seq data with a threshold of at least 90% coverage for CDS (**Supplementary Table 5**). In addition, we found that 41,127 of predicted proteins were homologous to genes identified in the NAM founder lines of maize[14] (**Supplementary Table 5**) and that 55.82% of the remaining 1,453 genes

were expressed in at least one of the 127 RNA-seq data sets we used (**Supplementary Table 14**) with a threshold of a FPKM (fragments per kilobase of transcript per million mapped reads) value larger than 1. In total, 1,029 predicted genes were newly anchored in the Mo17 chromosomes, including 246 newly assembled genes (**Table 1**) such as the *RPN8* gene (*Zm00014ba318810*, **Supplementary Fig. 15**), which its homolog in *Arabidopsis* encodes a 26S proteasome subunit that specifies leaf adaxial identity[15].

An extreme case of region with tandem gene duplications

One extreme case of region with tandem gene duplications was an approximate 800 kb newly assembled region between *Zm00014ba065330* and *Zm00014ba065630* on chr10, which contained a total of 29 genes and 6 putative pseudogenes. Of these 29 genes, 20 (mG1-1 to 1-20) were duplicated genes encoding threonine protein kinase, and 6 (mG4-1 to 4-6) were duplicated genes with unknown function (**Extended Data Fig. 9**). For the 6 pseudogenes, 2 of them (mPG2-1 and 2-2) were homologous with the 6 duplicated genes of unknown function, and another 4 (mPG1-1 to 1-4) were duplicated with each other (**Extended Data Fig. 9**). The corresponding region in the B73 genome (about 230 kb) was found to have 10 annotated genes. Among them, 3 genes (bG4-1 to 4-3) were homologous with the 20 duplicated threonine protein kinase genes, one gene (bG6) was homologous with the 6 duplicated genes of unknown function, and one gene (bG3) was homologous with the 4 duplicated pseudogenes in the Mo17 genome (**Extended Data Fig. 9**). Except for the genes mentioned above, the remaining 3 genes (mG2, 3, and 5) in the Mo17 genome and 5 genes (bG1, 2, 5, 7, and 8) in the B73 genome were not duplicated and no homology was found among them (**Extended Data Fig. 9**).

Variant distance between different satellite repeat copies

Totally, 122,760 intact knob180, 4,514 intact TR-1, and 44,747 intact CentC repeat copies were identified, respectively. Using the method reported previously[16], we generated a position probability matrix (PPM) for each type of satellites, and calculated a variant distance to the PPM for each satellite repeat copy. Substantial sequence variation was observed for knob180, TR-1 and CentC repeats, with a mean variant distance of 24.7, 54.7, and 13.1, respectively (**Extended Data Fig. 10a**).

Interestingly, there were two types of knob180 repeats according to their variant distances. About 20% of knob180 repeats displayed with relatively higher variant distances and were significantly enriched on Knob-8L (**Extended Data Fig. 10a and b**). These high varied knob180 repeats were not randomly distributed, but showed some extent of depletions across Knob-8L, with thousands (average about 4100, ranging from 1100 to 7900) of knob180 repeats between two adjacent depletions (**Extended Data Fig. 10c**). Satellite repeats with five or fewer pairwise variants were defined as higher-order repeat groups. A total of 3,695, 2,059, and 1,901 higher-order repeat groups, with an average of 13,155, 445, and 8,179 copies per group, were identified for knob180, TR-1 and CentC, respectively.

TEs enriched in 5S and 45S rDNA arrays

Among 343.8 kb TEs in the 45S rDNA array, 83.8% were Gypsy elements. By contrast, 81.3% of 116.9 kb TEs in the 5S rDNA array were Copia elements (**Fig. 4c and 4d**). Compared to the flanking regions, TEs inserted in the 5S rDNA array were enriched with Opie (29.9%) and ji (16.0%) families of Copia elements, while TEs in the 45S rDNA array were enriched with Prem1 (36.3%), Flip (21.5%), and Gyma (6.5%) families of Gypsy elements (**Supplementary Fig. 16**).

The correlation between gene number and non-CRM Gypsy abundance in centromeres

A total of 82 genes were identified in centromeres of the Mo17 genome. Most of these 82 genes were located in seven CentC-poor centromeres, including centromeres of chr2, chr3, chr4, chr5, chr6, chr8 and chr10 which harbored 13, 11, 4, 9, 15, 14 and 11 genes, respectively. By contrast, for the 3 CentC-rich centromeres, there was only 2 and 3 gene identified on centromeres of chr7 and chr9, respectively, and no gene was identified on centromeres of chr1 (**Fig. 5c**). Interestingly, more than half (ranging from 36.55% to 74.30%) of sequences of the 7 CentC-poor centromeres were occupied by non-CRM Gypsy, while the other 3 CentC-rich centromeres had only an average of 7.97% of non-CRM Gypsy (**Fig. 5a**). In contrast, no obvious difference in CRM abundance was observed between the two types of centromeres. In general, non-CRM Gypsy abundance was positively correlated (r = 0.722) with the number of genes in centromeres (**Supplementary Fig. 17b**), reflecting a potential role of

non-CRM Gypsy insertions in gene content in centromeres.

Supplementary Methods

ONT and PacBio libraries constructing and sequencing

For each ONT common sequencing library, approximately 3-4 μg of gDNA with size about 20 kb was selected using the Pippin HT system (Sage Science, USA). Next, the end reparation of DNA fragments and A-ligation reaction were performed using NEBNext Ultra II End Repair/dA-tailing Kit (Cat# E7546). Then, the adapter was ligated using ONT one-dimensional (1D) Sequencing Kit (SQK-LSK109). For each ultra-long Nanopore library, approximately 8-10 μg of gDNA with size about 100 kb was selected with SageHLS HMW library system (Sage Science, USA), and was then processed using ONT 1D Sequencing Kit (SQK-LSK109). ONT common and ultra-long sequencing libraries were all run on Nanopore PromethION sequencer.

PacBio libraries were constructed for sequencing according to PacBio's standard protocol. DNA damage repair, end repair and A-tailing, and hairpin adapters ligation were performed using the SMRTbell Express Template Prep Kit v2 (Cat# 100-939-900), followed by treatment of nuclease with SMRTbell Enzyme Cleanup Kits (PacBio) according to the instruction of Kits. Sequencing was performed on the PacBio SequeII platform with Sequencing Primer V2 and Sequel II Binding Kit 2.0. The raw data generated were processed using the CCS algorithm (v.4.0.4, https://github.com/pacificbiosciences/unanimity) with the parameter -minPasses3.

Closure of the TAG repeat array related gaps

The 5 TAG repeat array related gaps were manually closed based on the ultra-long ONT reads. To avoid the possible assembly errors around the boundaries of the gaps, the flanking 500 kb sequences of the two ends of each gap were removed from the basal Mo17 assembly, resulting in a trimmed Mo17 assembly. Then, the ONT data was iteratively mapped to the trimmed Mo17 assembly by Minimap2[17] with the parameters of '-x map -ont -r 10000'. For each round of mapping, the reads that could extend the gaps as far as possible were selected to extend the assembly of corresponding ends of gaps. The assembly extended by the reads was used for mapping in the next cycle. For each gap, the iteration of extension will be terminated when either the extension from two ends of the gap were overlapped, or no reliable reads could be found for extension from both ends. With this approach, gap1 and gap5

were closed.

Most regions of gap2, gap3, and gap_LCR6 were filled by ONT reads, but there was still a sub-gap in each of them that was not spanned by ONT reads. All these three sub-gaps were composed by TAG repeats longer than 90 kb. Next, we tried to determine if there were any other non-TAG sequences located inside of these three sub-gaps. Considering that the TAG repeats could be read as other microsatellites, we identified the ONT reads with the threshold that there was at least 5 kb microsatellite in one end of the read. A total of 4,129 quality-passed ONT reads longer than 100 kb were identified. Manual checking showed that except for 504 reads only composed by TAG repeats, all the remained 3,625 reads could be mapped back to our Mo17 assembly. This indicated that the three sub-gaps were all composed by TAG repeats only. Published BioNano molecules of Mo17 genome[4] were used to determine the lengths of these sub-gaps. BioNano molecules were mapped to the assembly by Solve (v3.5.1, https://bionanogenomics.com/support/software-downloads/) with default parameters (optArguments_nonhaplotype_noES_noCut_irys.xml). Based on the alignments, both sub-gap3 and sub-gap_LCR6 were spanned by BioNano molecules and thus their lengths were determined. Next, we tried to determine to length of sub-gap2. All 45.6× ONT (99.3 Gb) raw ONT reads longer than 150 kb were used to estimate the total length of 6 genomic regions with consecutive TAG repeats long than 90 kb, including the sub-gap2, as well as one 154.5 kb region in gap1, one 210.1 kb region in gap3, one 235.4 kb region and one 130.4 kb region in gap5, and one 128.5 kb region in Gap_LCR6. Two types of reads associated with these 6 regions were identified firstly. One type is the reads which could be mapped to the 6 regions. The second type is the reads which harbored with consecutive microsatellite repeats longer than 90 kb but could not be mapped to the 6 regions. Notably, in consideration of extra sequence errors for ONT reads with long TAG repeats and there was no other type of microsatellites longer than 90 kb in the genome, the reads which harbored with consecutive microsatellites longer than 90 kb, including microsatellites of non-TAG repeats, were also identified as the second type of reads. Then, we summed up the length of TAG repeats harbored for the two types of reads associated with these 6 regions, and normalized it by division by the average genome coverage of the data (45.6×), which resulted a total of 1,024.6 kb for the 6 regions. After subtracting the

lengths of the other 5 TAG repetitive regions with known sizes, the length of sub-gap in gap2 was estimated about 165.6 kb. Altogether, gap2, gap3 and gap_LCR6 was closed finally.

Closure of the 45S rDNA array related gap

The 45S rDNA array related gap was closed based on PacBio HiFi reads. We checked the direction of 45S rDNA sequences harbored by PacBio reads and ONT reads, and found there were two 'islands' with opposite directions of 45S rDNAs inside the gap. One island harbored with two rDNAs in which their intergenic spacer (IGS) regions were adjacent, whereas the other island harbored with two rDNAs in which their internal transcribed spacer (ITS) regions were adjacent. We performed the gap closure by extending along the transcriptional direction of 45S rDNAs. Therefore, three locations were served as starting points of extension, including the centromere-proximal end of the gap which 45S rDNAs direction was toward to the telomere, and the two sides of a 'island' with rDNAs which IGSs were adjacent with each other. Next, the extension was performed with following steps. Step 1: the IGS sequences of the 45S rDNAs to be extended were mapped to the PacBio reads including at least one intact 45S rDNA copy and intact IGS sequences of 45S rDNA adjacent to the 25S rRNA end of the intact 45S rDNA copy. BLASTN[18] (v2.9.0) was used for mapping with the parameters: -task megablast -max_hsps 1. Only the alignments in which the IGS sequences were mapped to the IGSs of the intact 45S rDNAs with more than 99% identity were retained. Step 2: For each of the three starting points, the best 5 hit reads were further selected based on the retained alignments in step 1, and were mapped back to the corresponding 45S rDNAs to be extended using BLASTN[18] (v2.9.0). The read with the best hit were selected for extension. Step 1 and step 2 were iterative performed by in-house script. If there were two or more reads matched the thresholds of this best-hit-based method, one of which will be randomly selected for extending. In addition, manual check and extension were needed when TE insertion happened or no PacBio reads were found for extension based on the thresholds set for this best-hit-based method.

Copy number estimation of rDNAs

The copy number of 5S and 45S rDNAs in the genome was estimated by the

blast-based method using both ONT ultra-long and PacBio HiFi data. The sequences of rDNA harbored on the data were identified by BLASTN[18] (v2.9.0) with the parameters: -task megablast -max_hsps 5000 -max_target_seqs 100000. The 5S rRNA monomer (Genebank ID: DQ351339.1), its1_5.8S rRNA_its2 sequences (Genebank ID: AF019817.1), 25SrRNA and 18SrRNA from Repbase[19] were used as the query sequences for BLAST. The total length of rDNA sequences identified was then normalized by division by the length of rDNA repeat unit and the average genome coverage of the data to estimate the copy number of rDNA in the genome.

The k-mer based method was used to estimate the copy number of 5S and 45S rDNAs in the genome with Illumina PCR-free data. The Illumina reads were aligned to the final assembly of the Mo17 genome with bwa mem[20], which default parameters for pair ends Illumina reads were used. Illumina reads aligned to the 5S and 45S rDNA regions were extracted from the aligned .bam files and then used for generation of 21 bp k-mers by jellyfish software[21]. Then, total length of 5S and 45S rDNAs in the genome were estimated according to an empirical formula: the total frequency of all k-mers / expected frequency of k-mer. The peak frequency of the k-mer frequency distribution was used as expected frequency of k-mer. Then, total length of 5S/45S rDNAs in the genome was divided by the length of single 5S/45 rDNA repeat unit to obtain an estimated copy number.

The copy number of 45S rDNAs in the genome was also estimated using Naica™ Crystal Digital PCR System (Stilla Technologies). Genomic DNA of Mo17 genome was isolated using CTAB method, and was then quantified using Qubit Flurometer with Qubit dsDNA HS Assay (Invitrogen). The digital PCR reactions for Zm00014ba171690, a single copy gene in the Mo17 genome, were run with 0.56 ng of gDNA. The digital PCR reactions for 45S rDNA were run with 0.056 ng of gDNA. Notably, the gDNA used for digital PCR was digested by MseI at 37℃ for 30 minutes at first. Digital PCR reactions were performed using the kit of PerfeCTa Multiplex qPCR ToughMix. Four technical replicates were set up. The sequences of specific primers and double-labelled probes (hydrolysis probe) designed were as follows: 45S rDNA, forward primer: 5’-ACTAGCCCCGAAAATGGATG-3’, reverse primer: 5’-CTACCACCAAGATCTGCACC-3’, probe:

5'-HEX-AAGCGCGCGACCCACACCCG-BHQ1-3'; Zm00014ba171690, forward primer: 5'-AACCCAGCTCGAAAAGTTGT-3', reverse primer: 5'-CGGATACAGAAGCAGGAGC-3', probe: 5'-CY5-CGCTCTCCGTTCGGGCGCG-BHQ2-3'.

Determination of the origin of the unmapped ONT reads

Except for properly mapped reads, fused reads, and symmetrical reads, the remained ONT reads were mapped to the maize organelle genomes (NCBI Genebank access ID: CM025451.1, CM025452.1, X86563.2, AY506529.1) using Minimap2[17] with the parameters of '-x map-ont -r 10000 -N 50'. The reads properly mapped to maize organelle genomes were identified using the same criteria for identifying reads properly mapped to the Mo17 nucleus genome. The remained unexplained reads were then mapped to the NCBI NT database (https://ftp.ncbi.nih.gov/blast/db/) by BLASTN[18] (v2.9.0) with the following parameters: -evalue 0.01 outfmt '6 qseqid qlen sseqid sgi slen pident length mismatch gapopen qstart qend sstart send evalue bitscore staxid ssciname' -task megablast. The reads which over 50% sequences were belong to hits not coming from plant were identified as microbial DNA contamination.

There were still 83,167 reads which were not explained by the above approaches. It is logical that if there reads were originated from assumed genomic regions not included in the T2T Mo17 assembly, they should can be supported by PacBio reads unmapped to the assembly. We aligned all 151.1 Gb Pacbio HiFi reads to the T2T Mo17 assembly using Minimap2[17] with the parameters of '-x map-pb -r 1000 -N 50'. About 4.78% PacBio HiFi reads were identified as unmapped reads with the threshold of the primary alignment and supplementary alignment (value of FLAG in SAM format file must be 0, 16, 2048, or 2064) with minimum query sequence coverage 0.85. The 83,167 unexplained ONT reads were averagely divided into 20 parts. Then, all unmapped PacBio HiFi reads were mapped to each part of unexplained ONT reads using Minimap2[17] with the parameters of '-x map-pb -r 1000 -N 50'. As the sequence coverage of quality-passed ultra-long ONT data was about 2.8-folds higher than that of PacBio HiFi data, a ONT read should be supported with about 7× coverages of PacBio reads if it was originated from assumed genomic regions not included in the assembly. Nearly 85% of these 83,167 ONT reads could not be mapped by any one of

PacBio reads. The remained 15% of these ONT reads had PacBio reads mapped but with low the coverage (average 38.1%) and depth (average 1.8×). Hence, we termed these 83,167 ONT reads as chimeric reads because there were no reliable PacBio HiFi read support.

Validation of the completeness of the T2T Mo17 assembly with PacBio and Illumina data

The completeness of the final T2T Mo17 assembly was estimated from mapped k-mers via Merqury[22] (v1.1). To eliminate the exogenous DNA contamination as much as possible, the completeness was analyzed by combining PacBio HiFi data and Illumina PCR-free data. In brief, the k-mer completeness of the Mo17 assembly was estimated through the ratio between the number of 'solid' k-mers in the Mo17 assembly and the number of 'solid' k-mers identified with both PacBio HiFi data and Illumina PCR-free data. Specifically, 'solid' here refers to k-mers which count were larger than 29 for PacBio HiFi data and larger than 30 for Illumina data, which were recommended by default parameters of Merqury. Notably, the reads that were originated from maize organelle genomes and the reads that were originated from microbial DNA contamination, which were determined according to the alignments with maize organelle genomes (NCBI Genebank access ID: CM025451.1, CM025452.1, X86563.2, AY506529.1) and the NCBI NT database (https://ftp.ncbi.nih.gov/blast/db/), were not used for analysis. A total of 598,833,512 solid k-mers were detected, of which 598,327,872 can be identified for the Mo17 assembly. Consequently, the completeness of the Mo17 assembly was estimated to be 99.92%. PacBio HiFi reads corresponding to the remained 0.08% k-mers were collected and then aligned to the final Mo17 assembly using Minimap2[17] with the parameters of '-x map-pb -r 1000 -N 50'. According to the alignments, the locations of these k-mers were determined. A k-mer originated from multiple locations and with frequency lower than 30 (the cut-off of solid k-mers) for each location was redefined as un-solid k-mer. For the k-mers with the frequency of at least one genomic location higher than 30, the read depths for corresponding genomic locations were checked. A k-mer was considered to be introduced by sequencing errors within reads and base errors harbored in the assembly if its corresponding read depth was normal (between 100 to 250, genome-wide average: 180.7).

Gene annotation

Both *ab initio* prediction and evidence-based prediction were used to predict the protein-coding genes in the Mo17 genome. Prior *ab initio* prediction, the repeat sequences were masked using RepeatMasker[23] (v4.1.1) with the Mo17 repeat library built by the Extensive de novo TE Annotator[24] (EDTA, v1.7.0). Then, Fgenesh[25] (v7.2.2) with the self-trained model parameters were run on the masked genome to predict gene models. For evidence-based prediction, four different approaches were performed, including RNA sequencing (RNA-seq) based prediction, ISO-seq based prediction, protein-based homology search, and evidence-based MAKER prediction.

For RNA-seq based prediction, RNA-seq data of Mo17 (see **Supplementary Table 14**) was firstly download from NCBI (https://www.ncbi.nlm.nih.gov/), and then low quality reads were filtered by FASTP[26] (v0.20.0) with the following parameters: '-q 3 -u 50'. Remained high quality reads of each RNA-seq were mapped to the T2T assembly of Mo17 genome using STAR[27] (v2.7.8a). The mapping results were then used for three genome-guided transcript assembly programs with default options, including StringTie[28] (v2.1.2), Cufflinks[29] (v2.2.1), and CLASS2[30] (v2.1.7). The GFF3 files of different RNA-seq data generated by the same transcript assembly program were merged and sorted by TACO[31] (v0.7.3) with default parameters. Mikado[32] (v2.0rc2) was used to obtain the optimal set of transcripts based on the following evidences: 1) the transcripts assembled by the three different transcript assembly programs; 2) high confidence set of splice junctions generated by Portcullis[33] (v1.2.0), which the mapped reads merged and sorted by SAMTools[34] (v1.9) were served as input; 3) ORFs identified for the assembled transcripts by TransDecoder[35] (v5.5.0); 4) transcripts homologous with SwissProt (plants, https://www.uniprot.org/) sequences as identified by Diamond[36] (v2.0.1). Default options were used for Portcullis and TransDecoder, while for Diamond[36], following parameters were set: --max-target-seqs 5 --outfmt 5. Overall, the input files for picking and annotating the optimal transcripts by Mikado included all transcript assemblies (with strandedness marked as True for all, and weights of Stringtie were set to 1) in GFF3 format, Portcullis generated splice sites in bed format, TransDecoder

results in bed format, homology results in XML format, and a scoring matrix in yaml format.

For ISO-seq based prediction, full-length cDNA data of Mo17 generated by sequencing of mixed RNA of seedling, root, silk, tassel, and bract were used. The gene models were predicted by PASA[37] (v2.3.3) with default options, with the step of aligning full-length cDNA data to the Mo17 genome using GMAP[38] (v.2017-11-15).

For protein-based homology search, we downloaded the predicted protein sequences of *Zea mays* (B73 AGPv4; Mo17 CAU1.0), *Sorghum bicolor* (NCBIv3), *Oryza sativa* (IRGSP1.0), and *Arabidopsis thaliana* (TAIR10) from http://gramene.org/. These protein sequences were aligned to the Mo17 genome using MMseqs[39] (v12.113e3) with default parameters. The result of alignment was then used for prediction of gene models by GeMoMa[40] (v1.6.4). First, introns were extracted by GeMoMa module ERE (Extract RNA-seq Evidence) from the mapped RNA-seq reads. Next, the module GeMoMa was run to build gene models for each reference species by combining the result of protein alignment and extracted intron information. Gene predictions from different reference species were then combined and filtered by GeMoMa modules GAF and AnnotationFinalizer to obtain a final annotation.

For evidence-based MAKER prediction, MAKER[41] (v 2.31.10) was used to predict gene models, by combined with protein homology evidence of *Zea mays* (B73,Mo17), *Sorghum bicolor*, *Oryza sativa*, *Arabidopsis thaliana*, and SwissProt (plants) database (https://www.uniprot.org/uniprot/), as well as transcript evidence generated by RNA-seq and ISO-seq mentioned above.

Gene models predicted by GeMoMa (homology evidences based), Mikado (RNA-seq data based), PASA (ISO-seq data based), MAKER (EST/Homology evidences based), Fgenesh (*ab initio* predicted) programs were combined by EVidenceModeler (EVM, v1.1.1) [42] to generate a non-redundant set of gene annotation. Weight for each type of origins was set as follows: PASA (10) = Mikado (10) > GeMoMa (8) > MAKER (5) > Fgenesh (1). For the normal operation of the program, the format of gff3 file generated by GeMoMa was changed. Because the result of gene models integrated by

EVM do not have 5' and 3' untranslated regions (UTRs) and alternative splicing information, we then used PASA[37] (v2.3.3) to update the gene models resulted after EVM integration with an iterative 2-pass. The transcripts in FASTA format generated by Mikado were used for the first round, and the Mo17 full-length cDNA file was used in the subsequent round. All predicted gene models were annotated by InterProScan[43] (v5.39-77.0) with gene ontology (GO) annotation pipeline, which was ran with the parameters of '-f tsv -iprlookup -goterms -dp'. Transposable element (TE) related genes were filtered by the corresponding InterPro entry. The remained genes were termed as high-confience protein coding genes.

Supplementary Notes

Repeat unit sequences of three newly identified satellite repeats

sat268,

5’-AATAGTAGGCATCGTAGAGAAAACCGTAGCGGGCAGTTGAGTTGTTTCC
GTAATTAAAAAAATATTTTGCTGTTTTTTGGATTTTTTTGTGATTCTTAATTTG
TCGAGGACACTCGGCAAAAAATGTGAGTCCGGTAGTGAATCACCCCACAC
CTGTACGCGCAACGAGATTCTTCACGAAGCACGCGCGAACAAGTGGAAGA
CGAAGCACGAAACTAGCGCGACGTCGTAGTGTCCCCCTCAGCTGGGAGAG
AGAAGCTTGCGACCA-3’.

sat261,

5’-ACCTCGGCGGGCCCTGCATGCCTTGGCTGGATCCACCGCGAAAACCTAG
CCGCCTGCCCTCCTCCGCCGCGGCCGGGACTTGTGAGAAACTCAGATGCC
GTTAATCAACCGCCGCCAACGACGAGGAGACCCTTGCGAGCACGGCCATC
ATCCGTCCGCGACCGAGGCGCGCCCGCGCGCGGCGGCGGCGCGGCAAGGC
GTCGCACCCTTGGAATAGTTTCTTCGGAAAGCGACCCACTGATCAATAGGA
GTACAAGGTTT-3’.

sat112,

5’-GTGGGCATTGTAGGGTTCGTCCGAAACGCAGCAAAACACGTGGGACGA
CCGATCCACGTCAAAAGGAGGGAGAGTGGGCATTCTAGGGTTCGTCCGAA
ACGCAGCAAAACAC-3’.

The sequences of the two subtelomeric repeat in the Mo17 genome

Subtelomeric repeat 1,

5’-ATGCCACCCGTTCGCCACCCTTGTTTTGGCCACTAAGACAGGTAAGGTT
GTTTTTGGCCTCGCGTGAGCTACAACACATGTTTTCATGGCCGAACAACCA
ATTTAGTGTCCAACCATAGTACACTAGTGTTCAAACCATAGTACACATTTTT
GTCCCCGGAGGCCTGTAAGGCTATTTTTGGCCTCCCGCGACACATGTTTTCT
TCGTCAAACAACAATTTCATGCCTCCCGCCCGCCAAACATGTTTTGGCTACT
GACATGGGTAAGGTTGTTTTTAGCATCTGTTGAGCTACATCACACAAAACA
CTTAAATCCTAAACACCGAGCCCCAAACCCTAAACCATGAACCCGGAACCG
CGAACCCTTTGACTAAAACCCGACCCCCAAAACACAAAATCACAAATCCC
AAACTCCAAACCCTAAACATTAAACCCCAAACCCTAACCCTCAAATCAAAA
CCCAAAATCTCGAATCCCAAAAGCCTAATCTCTAAACCCCGAGCCCCAAAC

CCTAAAGCCTAAACATTGAACCCCAAACCCTAATTTGTGAACCCTATACCTC
GAACCCTAAAACAAAGACCCGACCACCAAAACACAAAACTCTAAACCCCT
AACTCCAAACCCTAAAA-3’.

Subtelomeric repeat 2,

5’-CACGTTTTGGTCCCCGGAGGCCGGTAAGGCAATTTTTGGCCTCCCGTGA
CACATGTTTTCATCGTCAAACAACGGTTTCATGCCTCCCGTCCGCCACCCAT
GATTTGGCCACTGAGACTGCTAAGGCTGTTTATGGCCTCCCGTAAGCTATAG
CACACGTTTTCATGGTCGAGCGACCATTTTTATGTACGTGTTCCACCACCCG
CGTTTTGGTCCCCAAAGTACCTTAAAGTTGTTCTTGGTCTCCCACGAGCTGT
AGCACACGTTTCCGAGGCCAAAGAGCTAATTTCATGTATCCGACCTGCCAC
CTATGTTTTAGACCGGAGAGGCCGTTACGACATTTTTTTGCCACAAGTGAG
CTATAGCACACATTTTTATGGTAACCTAGACCCCGAATCCAATCCCTAAACC
CTAACCTTAAACACCAAAACTAAAAGGTTTTAGTGTCCAAACCATAGTAAA
ATGAAGTTTGAGTCTCCAAACCATAGTAAGCTTGCAGGCATGGTAGAATTTT
AGTGTCCAAACCAAAGTA-3’.

Supplementary Figures

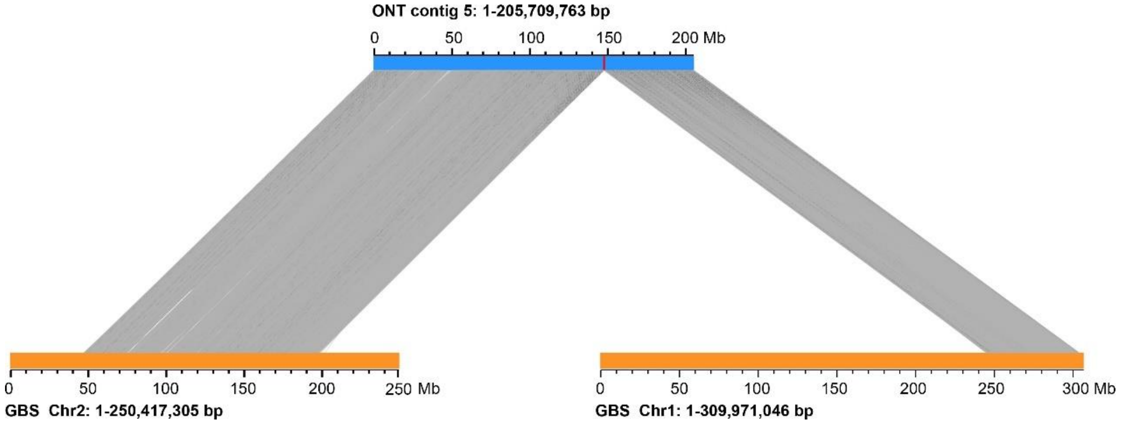

Supplementary Fig. 1. Schematic showing an initial ONT contig containing both the regions of chromosomes 1 and 2 due to assembly error. The incorrect assembly is introduced by the complex and long TAG repeat arrays existed on chromosomes 1 and 2. The contig was spilt into two contigs, corresponding to contig3 and contig6 of the basal Mo17 assembly. The misassembled region related to two TAG array related gaps on chromosome 1 (Gap 2) and 2 (Gap 5) was represented by the red box.

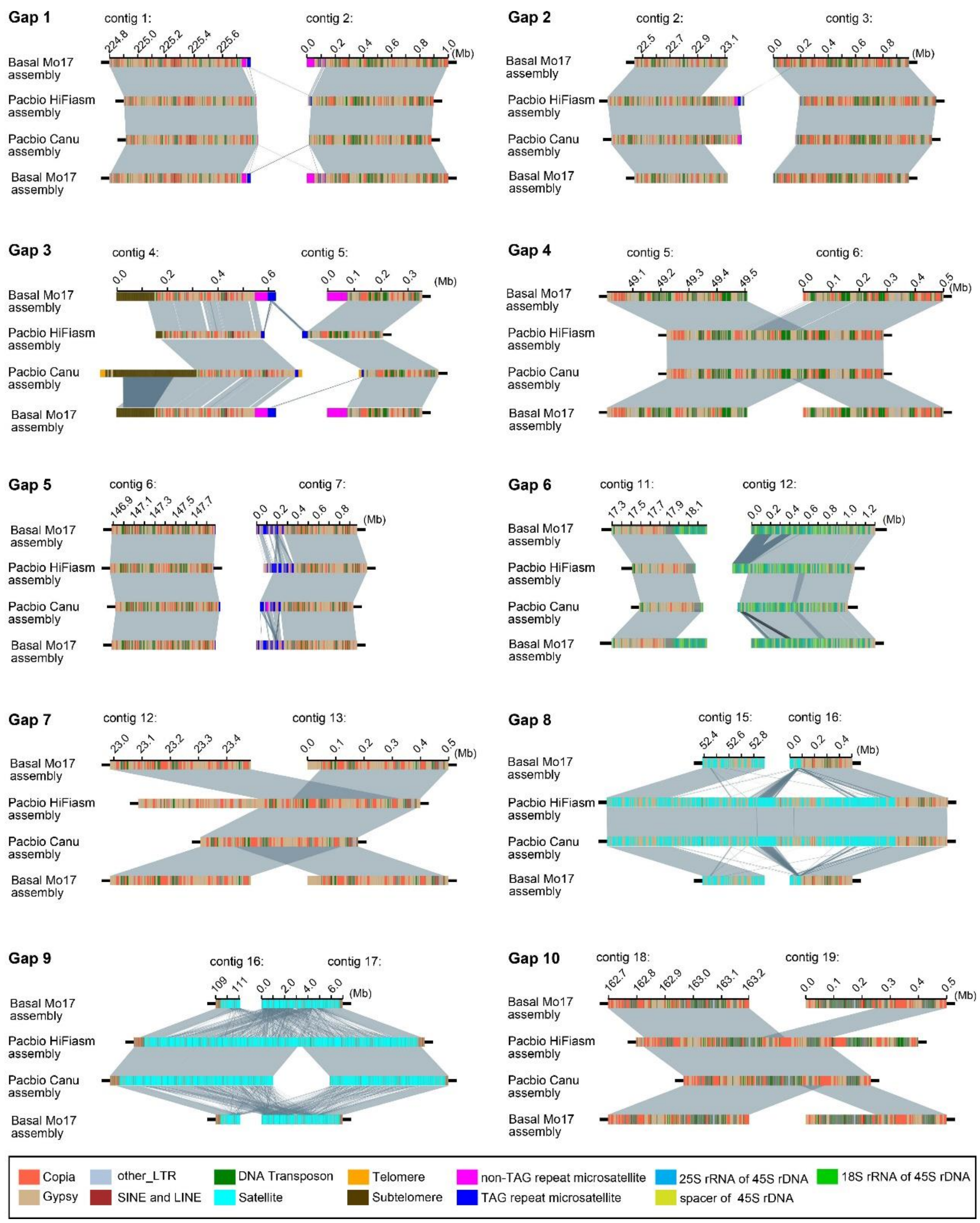

Supplementary Fig. 2. Comparison of the assembly of the flanking regions of 10 gaps on the basal Mo17 assembly with PacBio assembly. According to the alignment between the basal Mo17 assembly and PacBio assembly, five of 10 gaps (gap4, 7, 8, 9 and 10) on the basal Mo17 assembly could be closed by PacBio Hifiasm and/or Canu assemblies.

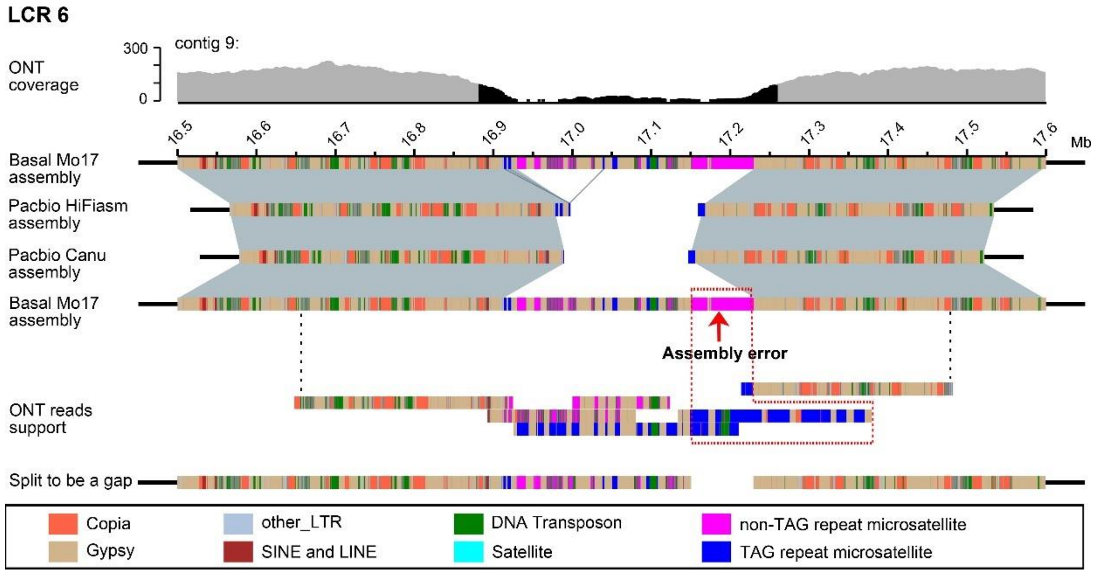

Supplementary Fig. 3. Schematic showing the assembly error of a TAG repeat array related LCR on chromosome 4. According to the mapping of ONT reads, there was obviously assembly errors for the LCR6 around 17.2 Mb of chromosome 4, which was then spilt and thus introduced a new gap (termed as gap_LCR6). Black shades refer the regions with reads depth lower than 100.

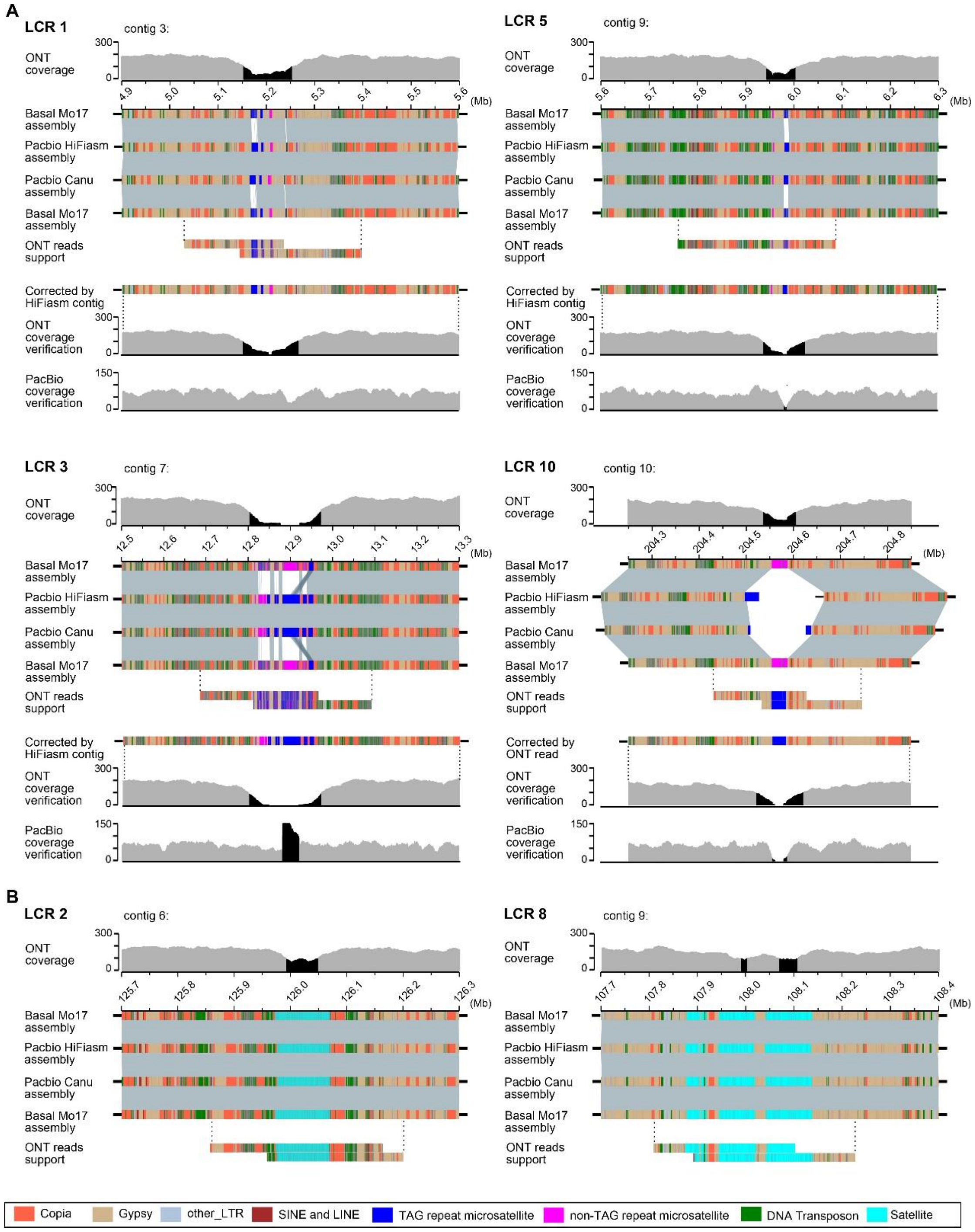

Supplementary Fig. 4. Correction of the assembly of LCRs without structural errors. a) Correction of the assembly of the 4 TAG repeat array related LCRs. Black shades refer local coverage-anomalous regions. According to the alignment with the PacBio assembly and tiling ONT reads, there were no large structural errors existed for the 4 LCRs on the basal Mo17 assembly. Notably, in the final Mo17 assembly, the sequence errors introduced by extra sequence errors for ONT reads with long TAG repeats were corrected by corresponding PacBio assembly (LCR1, 3, and 5) or

corresponding ONT reads which TAG repeats was not miscalled as other microsatellites (LCR10). b) The assembly of the sat268 (left panel) and Cent4 (right panel) related LCRs observed. According to the alignment with the PacBio assembly and tiling ONT reads, there were no large structural errors existed for the 2 LCRs on the basal Mo17 assembly.

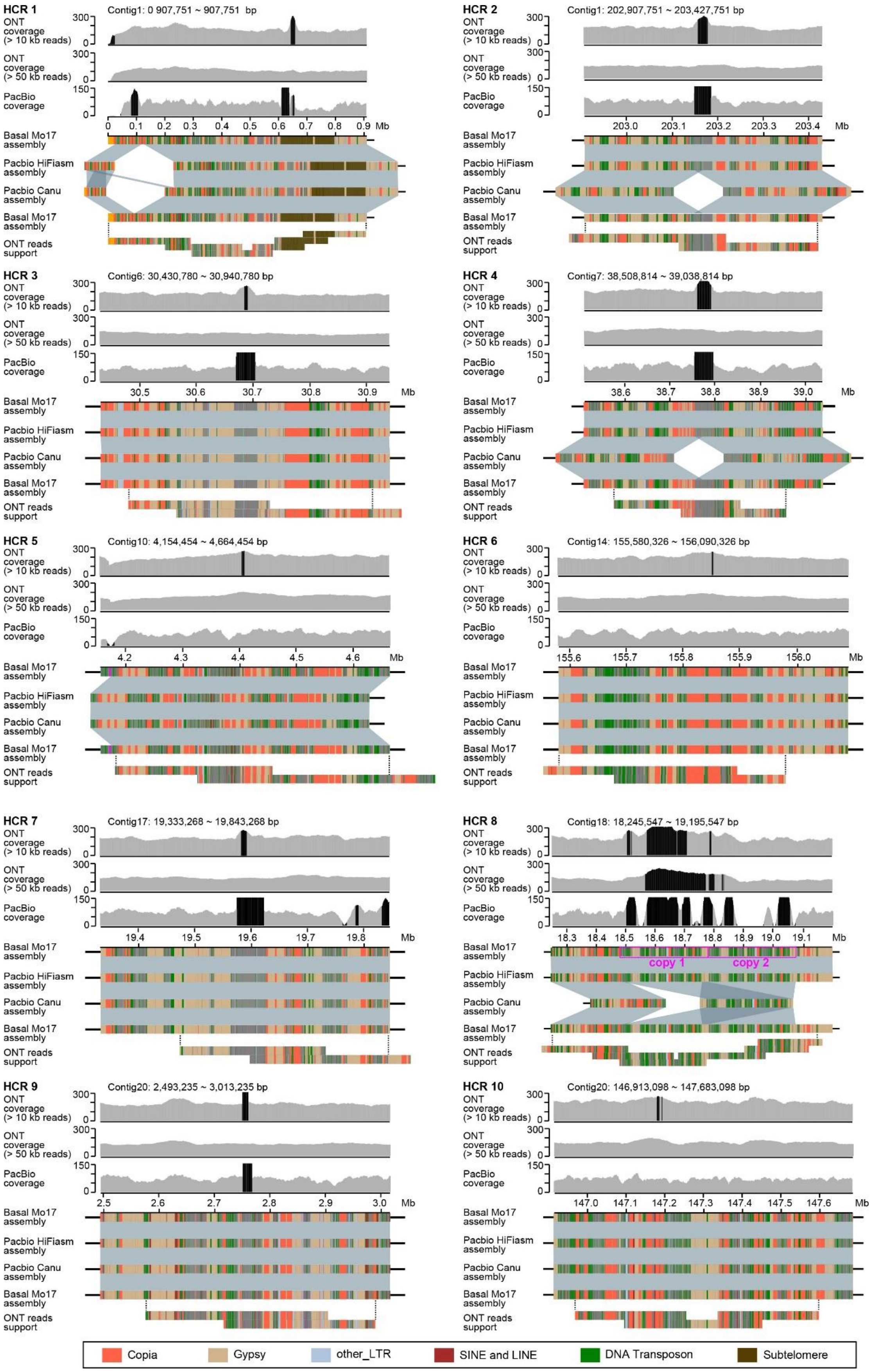

Supplementary Fig. 5. Validation of the assembly of high coverage regions in the final T2T Mo17 assembly. Detailed displaying of high coverage regions (HCRs) with

ONT reads depth higher than 250 for the T2T assembly of Mo17 genome, which ultra-long ONT reads longer than 10 kb were used for analysis. HCR1 was related to subtelomeric repeats. HCR5, 6, 8, and 10 were related to TEs. HCR2, 3, 4, 7 and 9 were related to genomic regions homologous with maize mitochondrion genome (**Supplementary Fig. 7)**.

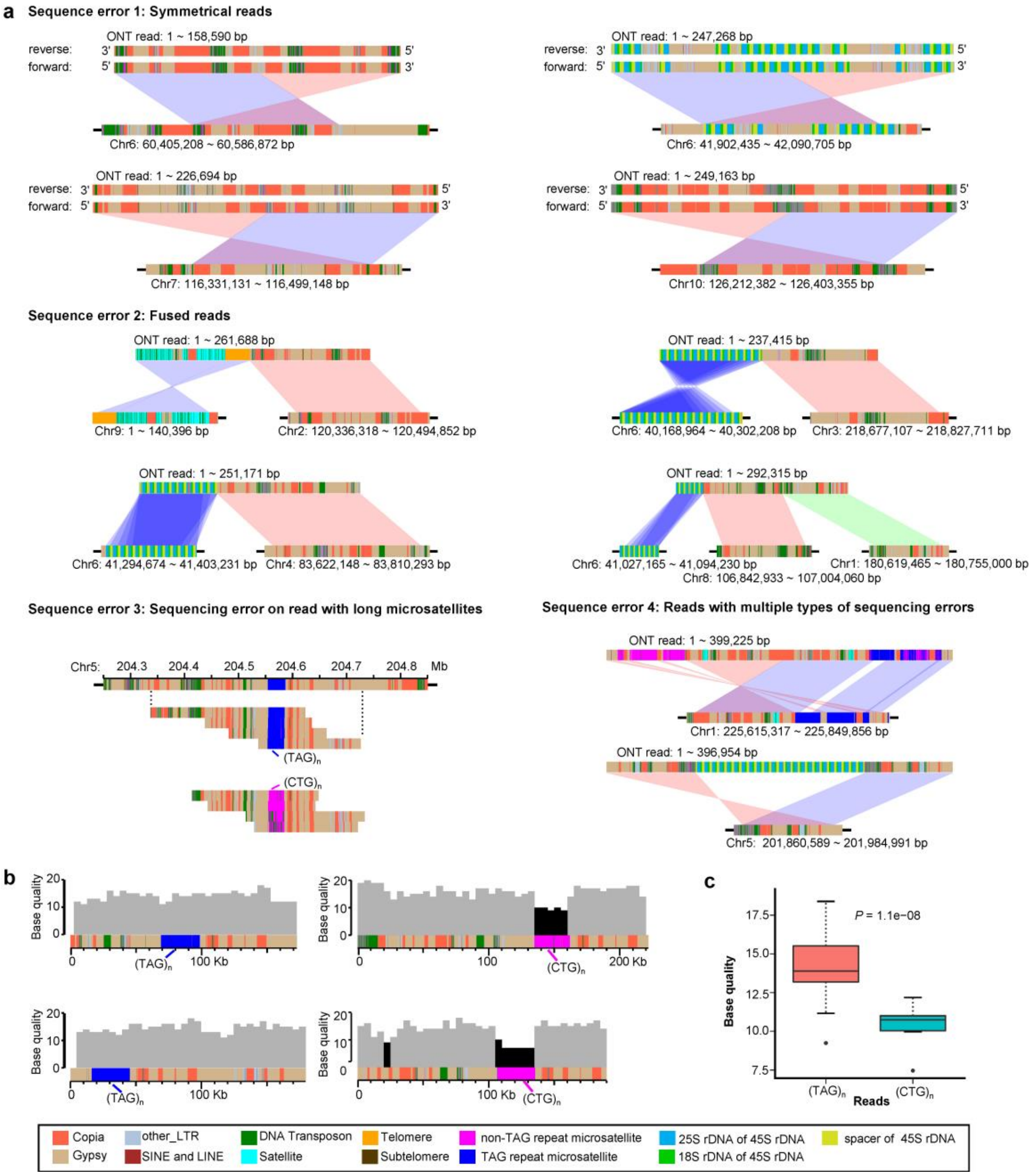

Supplementary Fig. 6. Sequencing errors of ONT reads. a) The examples of four types of ONT reads with sequencing errors. The example of sequence error 3 is correspond to LCR10 in the basal assembly. b) Base quality of four reads corresponding to LCR10. c) The base quality of reads corresponding to LCR10. Only the quality of bases in microsatellite region were analyzed. The number of analyzed reads which microsatellites were called as TAG repeats and CTG repeats were 31 and 15, respectively. In box plots, the 25% and 75% quartiles are shown as lower and upper edges of boxes, respectively, and central lines denote the median. The whiskers extend to 1.5 times the interquartile range. Data beyond the end of the whiskers are

displayed as black dots. *P*-value was reported from two-tailed t-test without adjustment for multiple comparisons.

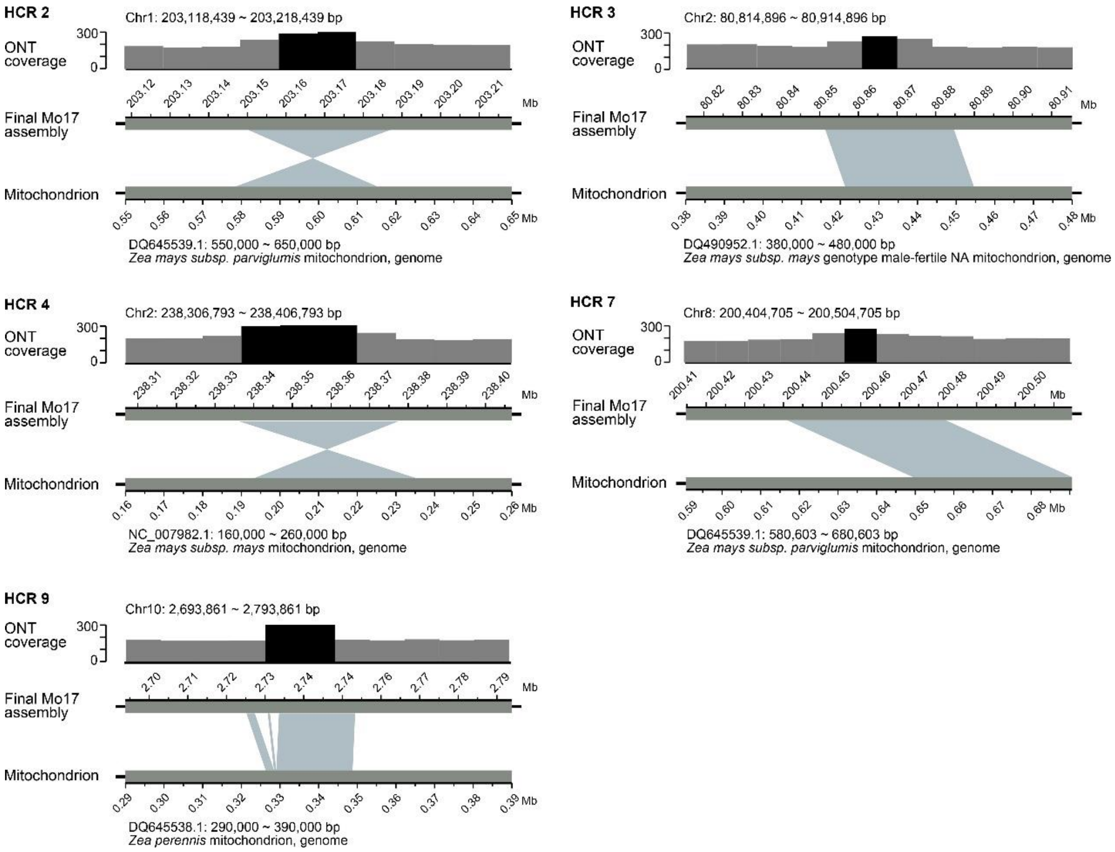

Supplementary Fig. 7. Five high coverage regions in the final T2T Mo17 assembly were related to genomic regions homologous with maize mitochondrion genome. DQ645538.1, DQ645539.1, DQ490952.1, and NC_007982.1 were the NCBI access numbers for corresponding mitochondrion genomes. The identity of all alignments showed were higher than 99%. Genomic regions with depth higher than 250 are shown in black shades.

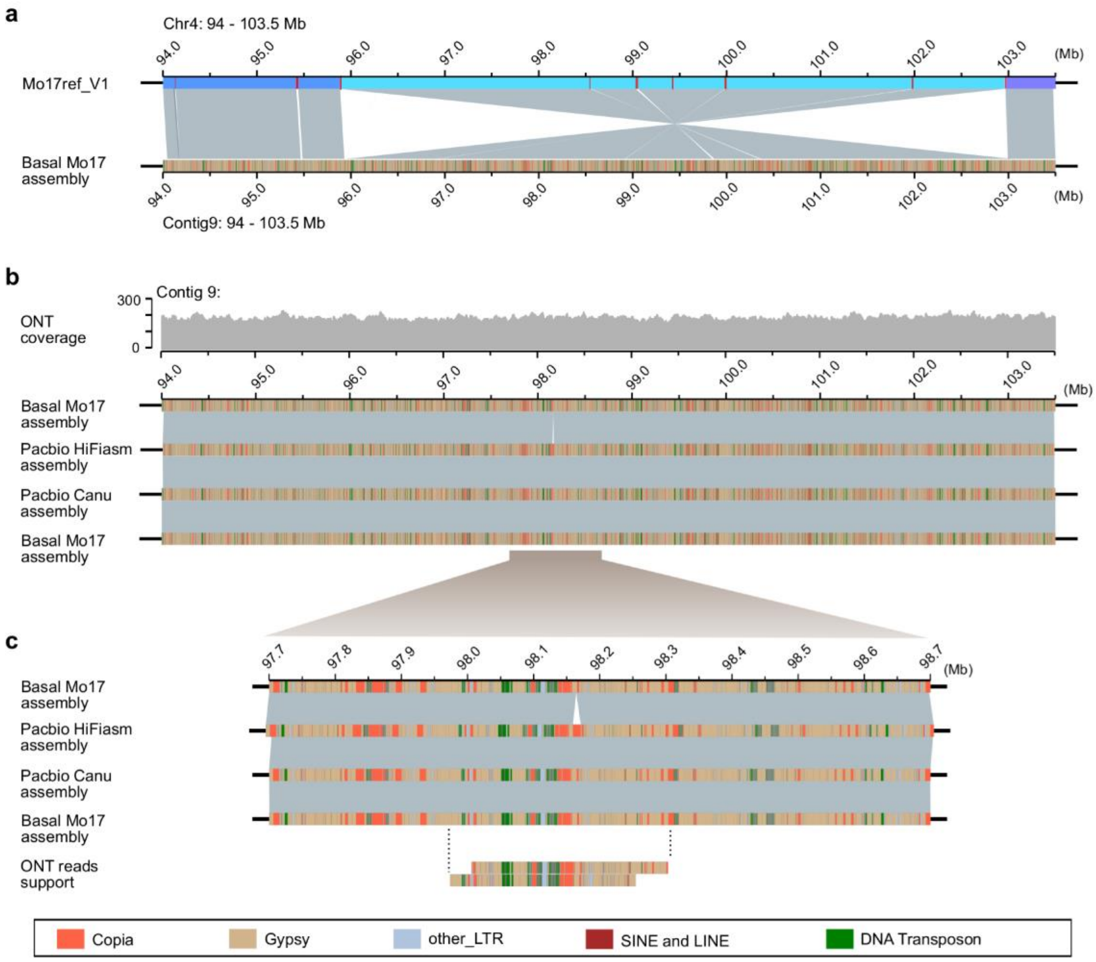

Supplementary Fig. 8. Validation of the assembly of an inversion between the basal Mo17 assembly and the Mo17ref_V1. a) An inversion around 96 to 103 Mb on chromosome 4 was observed between the basal Mo17 assembly and the Mo17ref_V1. For the Mo17ref_V1, different colored blocks refer the scaffolds, and the red blocks refer the gaps. b) The validity of the basal Mo17 assembly at this region was validated by concordant PacBio assembly and uniform ONT reads coverage, which suggested that the inversion was introduced by the anchor and orient errors of the contigs of Mo17ref_V1. c) We noted that there was an indel about 15 kb at 98.15 to 98.17 Mb of contig 9 of the basal Mo17 assembly as compared with PacBio HiFiasm assembly. The validity of the basal Mo17 assembly at this region was validated by concordant PacBio Canu assembly and tiling ONT reads, which suggested that the indel was introduced by assembly error of corresponding contig of PacBio Hifiasm assembly.

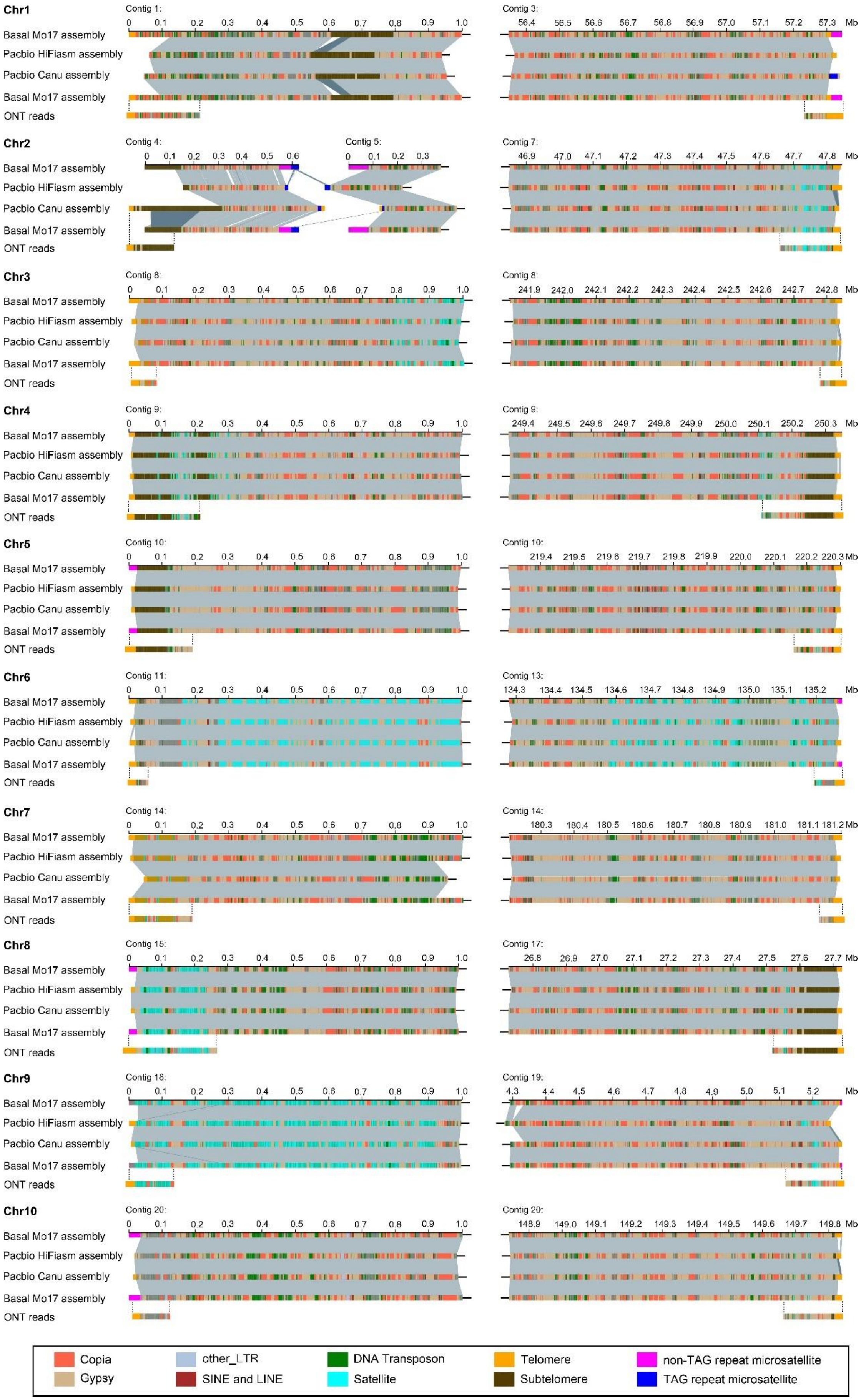

Basal Mo17 assembly
Pacbio HiFiasm assembly
Pacbio Canu assembly
Basal Mo17 assembly
ONT reads
Chr1
Contig 1:
0 0.1 0.2 0.3 0.4 0.5 0.6 0.7 0.8 0.9 1.0
Contig 3:
56.4 56.5 56.6 56.7 56.8 56.9 57.0 57.1 57.2 57.3 Mb
Chr2
Contig 4:
0 0.1 0.2 0.3 0.4 0.5 0.6
Contig 5:
0 0.1 0.2 0.3
Contig 7:
46.9 47.0 47.1 47.2 47.3 47.4 47.5 47.6 47.7 47.8 Mb
Chr3
Contig 8:
0 0.1 0.2 0.3 0.4 0.5 0.6 0.7 0.8 0.9 1.0
Contig 8:
241.9 242.0 242.1 242.2 242.3 242.4 242.5 242.6 242.7 242.8 Mb
Chr4
Contig 9:
0 0.1 0.2 0.3 0.4 0.5 0.6 0.7 0.8 0.9 1.0
Contig 9:
249.4 249.5 249.6 249.7 249.8 249.9 250.0 250.1 250.2 250.3 Mb
Chr5
Contig 10:
0 0.1 0.2 0.3 0.4 0.5 0.6 0.7 0.8 0.9 1.0
Contig 10:
219.4 219.5 219.6 219.7 219.8 219.9 220.0 220.1 220.2 220.3 Mb
Chr6
Contig 11:
0 0.1 0.2 0.3 0.4 0.5 0.6 0.7 0.8 0.9 1.0
Contig 13:
134.3 134.4 134.5 134.6 134.7 134.8 134.9 135.0 135.1 135.2 Mb
Chr7
Contig 14:
0 0.1 0.2 0.3 0.4 0.5 0.6 0.7 0.8 0.9 1.0
Contig 14:
180.3 180.4 180.5 180.6 180.7 180.8 180.9 181.0 181.1 181.2 Mb
Chr8
Contig 15:
0 0.1 0.2 0.3 0.4 0.5 0.6 0.7 0.8 0.9 1.0
Contig 17:
26.8 26.9 27.0 27.1 27.2 27.3 27.4 27.5 27.6 27.7 Mb
Chr9
Contig 18:
0 0.1 0.2 0.3 0.4 0.5 0.6 0.7 0.8 0.9 1.0
Contig 19:
4.3 4.4 4.5 4.6 4.7 4.8 4.9 5.0 5.1 5.2 Mb
Chr10
Contig 20:
0 0.1 0.2 0.3 0.4 0.5 0.6 0.7 0.8 0.9 1.0
Contig 20:
148.9 149.0 149.1 149.2 149.3 149.4 149.5 149.6 149.7 149.8 Mb
Copia
Gypsy
other_LTR
SINE and LINE
DNA Transposon
Satellite
Telomere
Subtelomere
non-TAG repeat microsatellite
TAG repeat microsatellite

Supplementary Fig. 9. Validation of the assembly of terminal 1 Mb regions for the chromosomes of the basal Mo17 assembly. The assembly of terminal 1 Mb regions for 10 chromosomes of the basal Mo17 assembly were validated by comparing with PacBio assembly. In consideration of that the telomeric repeats can be read as other sequences due to the extra sequence errors of ONT reads, the ONT reads with telomeric repeats were also used to validate the assembly of telomeric regions.

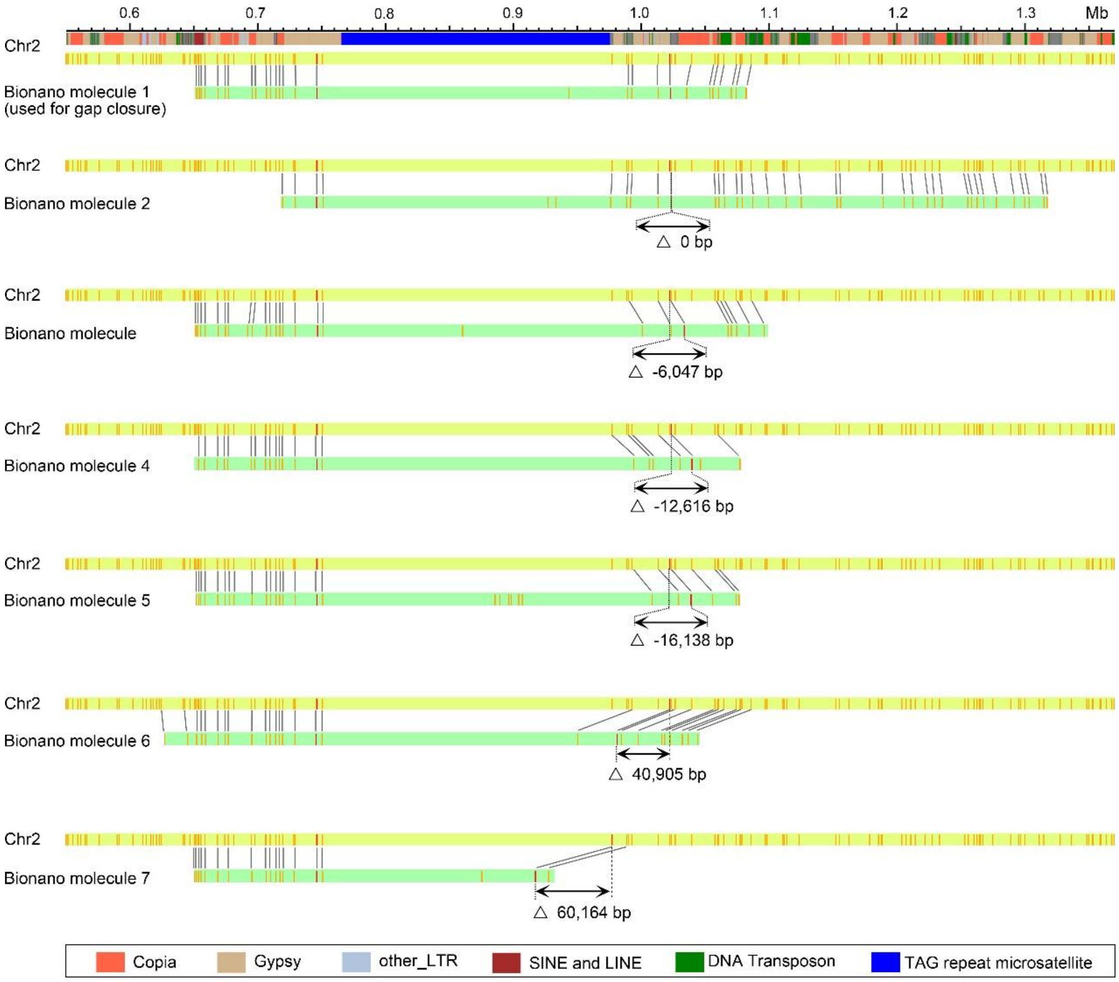

Supplementary Fig. 10. Alignments of BioNano molecules with a TAG repeat array on chromosome 2. The TAG repeat array was corresponded to gap3 in the basal Mo17 assembly. The length differences between the assembly and BioNano molecules might reflected the length variation of the TAG repeats. The positive and negative value indicated that the assembly length was longer and shorter than that estimated by corresponding BioNano molecules, respectively. The value of the length difference was calculated based on the restriction enzyme cutting sites which were marked as red.

a

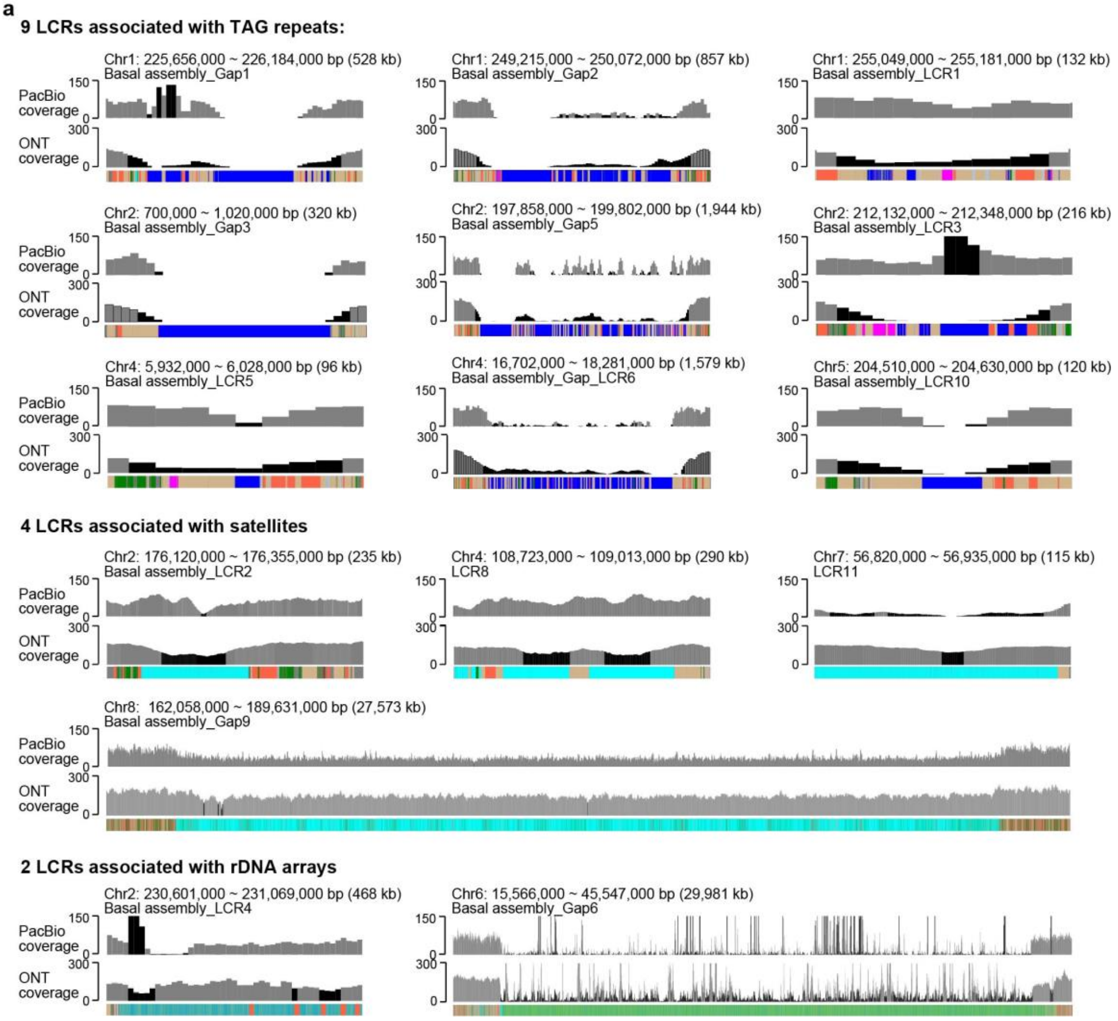
9 LCRs associated with TAG repeats:
Chr1: 225,656,000 ~ 226,184,000 bp (528 kb)
Basal assembly_Gap1
Chr1: 249,215,000 ~ 250,072,000 bp (857 kb)
Basal assembly_Gap2
Chr1: 255,049,000 ~ 255,181,000 bp (132 kb)
Basal assembly_LCR1
Chr2: 700,000 ~ 1,020,000 bp (320 kb)
Basal assembly_Gap3
Chr2: 197,858,000 ~ 199,802,000 bp (1,944 kb)
Basal assembly_Gap5
Chr2: 212,132,000 ~ 212,348,000 bp (216 kb)
Basal assembly_LCR3
Chr4: 5,932,000 ~ 6,028,000 bp (96 kb)
Basal assembly_LCR5
Chr4: 16,702,000 ~ 18,281,000 bp (1,579 kb)
Basal assembly_Gap_LCR6
Chr5: 204,510,000 ~ 204,630,000 bp (120 kb)
Basal assembly_LCR10
4 LCRs associated with satellites
Chr2: 176,120,000 ~ 176,355,000 bp (235 kb)
Basal assembly_LCR2
Chr4: 108,723,000 ~ 109,013,000 bp (290 kb)
LCR8
Chr7: 56,820,000 ~ 56,935,000 bp (115 kb)
LCR11
Chr8: 162,058,000 ~ 189,631,000 bp (27,573 kb)
Basal assembly_Gap9
2 LCRs associated with rDNA arrays
Chr2: 230,601,000 ~ 231,069,000 bp (468 kb)
Basal assembly_LCR4
Chr6: 15,566,000 ~ 45,547,000 bp (29,981 kb)
Basal assembly_Gap6
PacBio coverage
ONT coverage
150
0
300
0

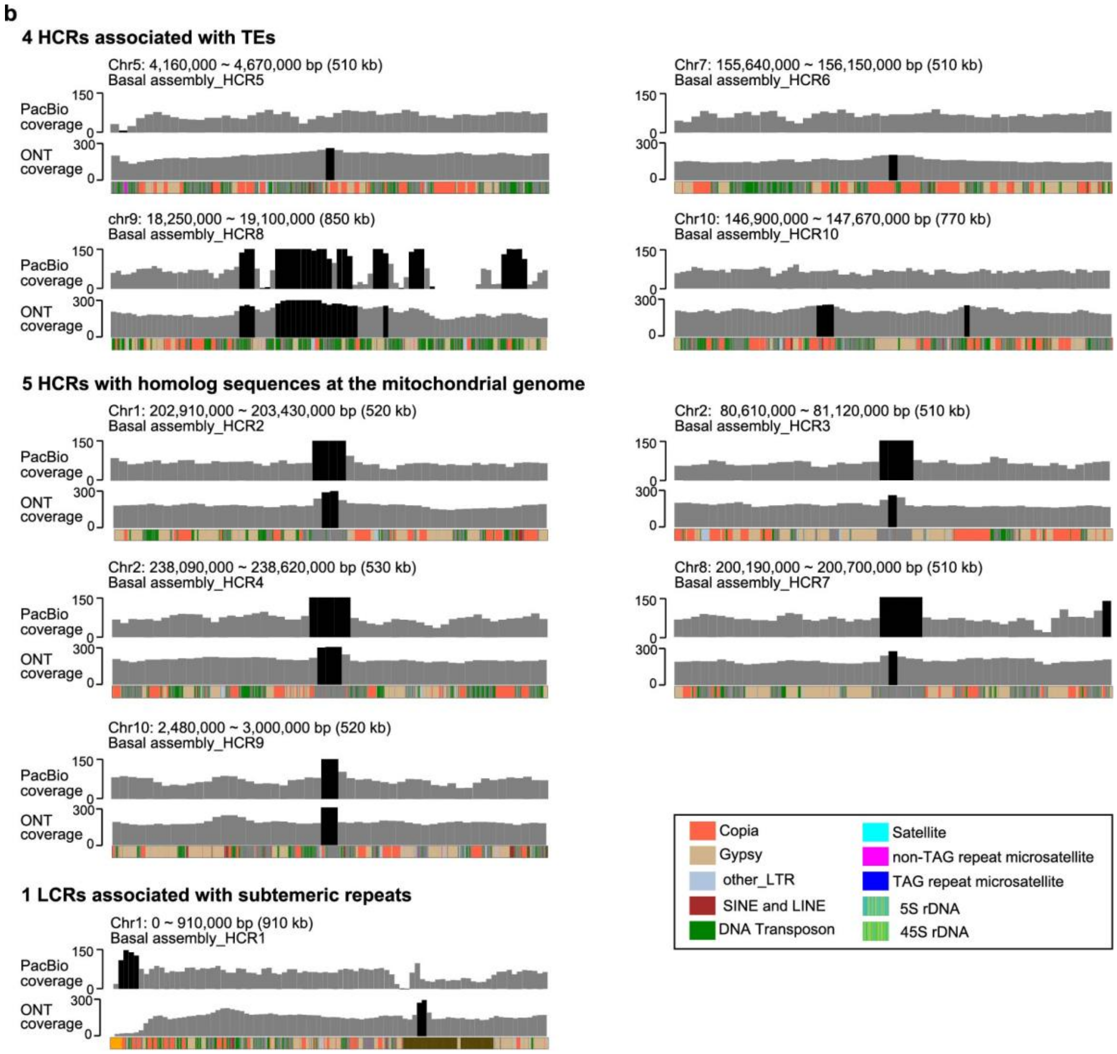

Supplementary Fig. 11. The LCRs and HCRs identified on the final T2T Mo17 assembly. a) and b) Detailed displaying of the LCRs (a) and HCRs (b) of the final T2T Mo17 assembly identified based on the ONT reads, which corresponding regions on the basal assembly were referred. The coverage of terminal 1 Mb regions of chromosomes was showed on **Extended Data Fig. 4.**

a

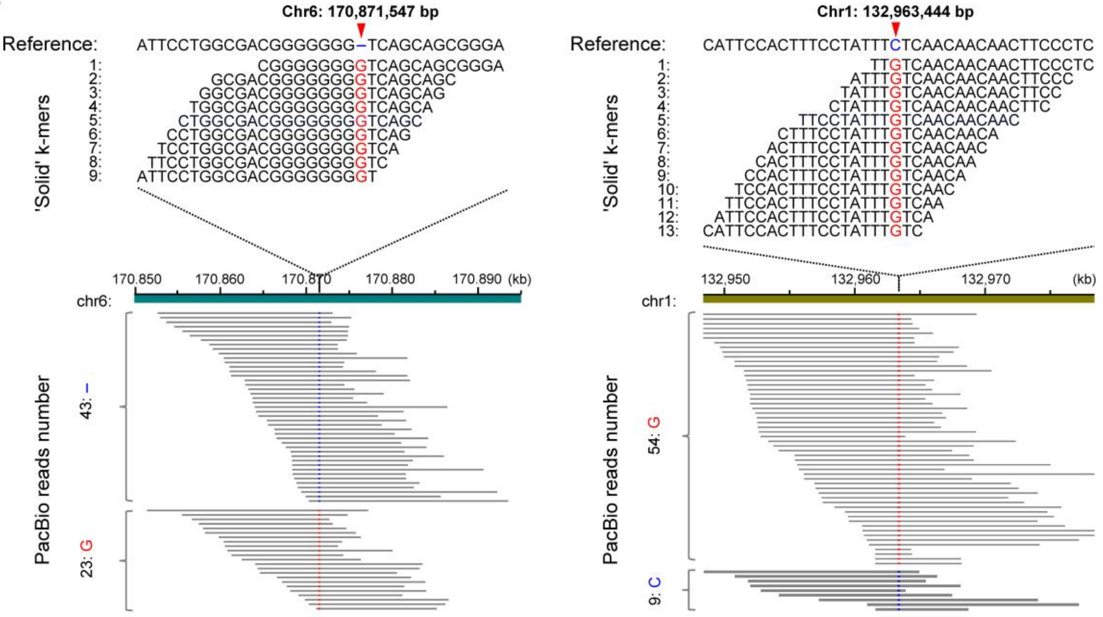

b

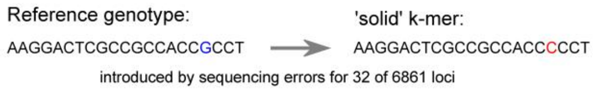

NO.	Chr	Position (bp)	Reads number (G/C)	NO.	Chr	Position (bp)	Reads number (G/C)
1	1	30,979,550	27/4	19	5	24,770,350	33/3
2	1	90,760,176	38/4	20	5	58,670,948	33/3
3	1	112,917,167	40/4	21	5	87,790,485	29/3
4	1	153,062,212	34/4	22	5	150,491,008	38/4
5	2	34,309,489	30/3	23	5	217,371,314	32/5
6	2	54,467,051	31/3	24	6	116,652,500	30/3
7	2	67,511,823	32/3	25	7	4,635,636	44/4
8	2	214,969,791	39/3	26	7	144,860,437	34/3
9	2	221,508,128	31/3	27	8	147,589,476	34/3
10	2	246,510,303	31/3	28	8	162,532,223	41/4
11	3	190,913,431	40/4	29	9	99,619,143	31/3
12	3	193,002,765	45/4	30	9	125,580,031	33/3
13	3	200,218,801	46/5	31	10	10,516,824	37/4
14	3	219,313,364	34/5	32	10	11,494,086	36/4
15	3	233,740,142	34/3	33	10	18,445,580	34/4
16	4	22,651,876	34/3	34	10	101,612,151	34/4
17	4	108,114,970	33/4	35	10	116,766,102	38/4
18	4	245,796,842	33/3	36	10	139,943,470	30/3

Supplementary Fig. 12. Examples of solid k-mers unincluded in the Mo17 genome. a) Examples of 'solid' k-mers unincluded in the Mo17 genome, which was introduced by base errors within reads (left) and assembly (right). b) An example of 'solid' k-mer unincluded in the Mo17 genome, which was introduced by sequencing errors of reads originated from different genomic regions. PacBio HiFi reads were used for analysis.

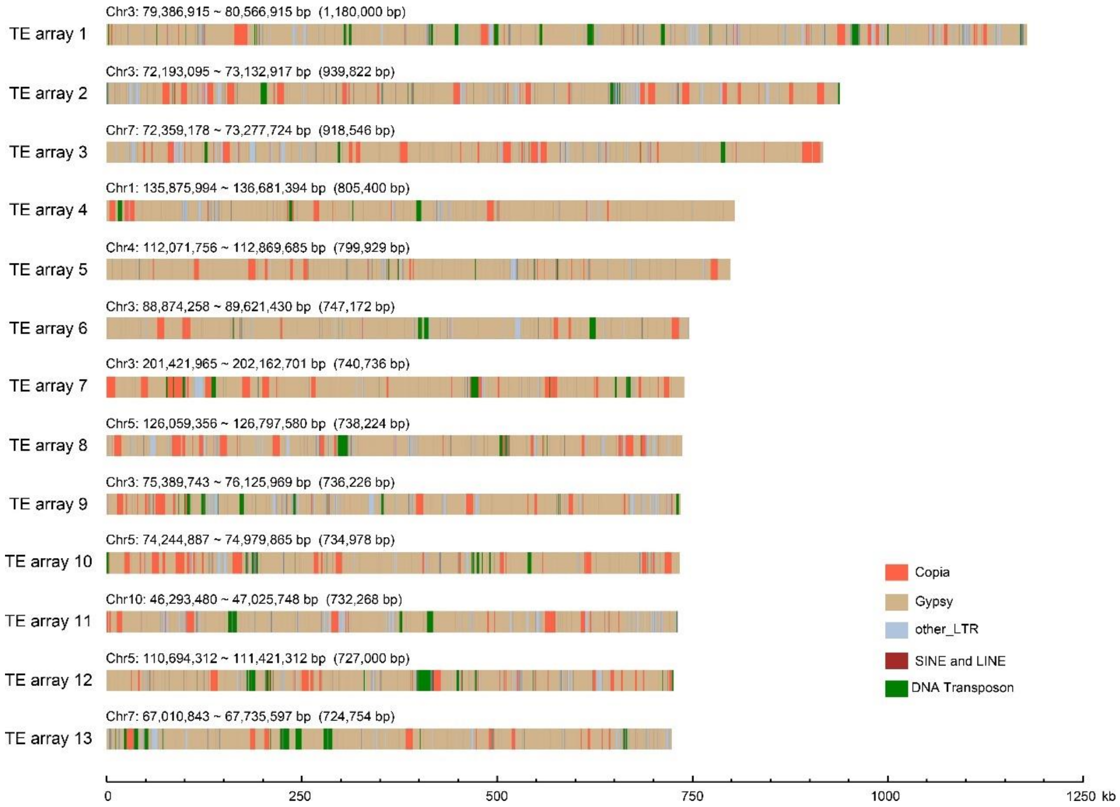

Supplementary Fig. 13. TE arrays larger than 700 kb.

There was no gene in these TE arrays, which more than 95% sequences were TEs.

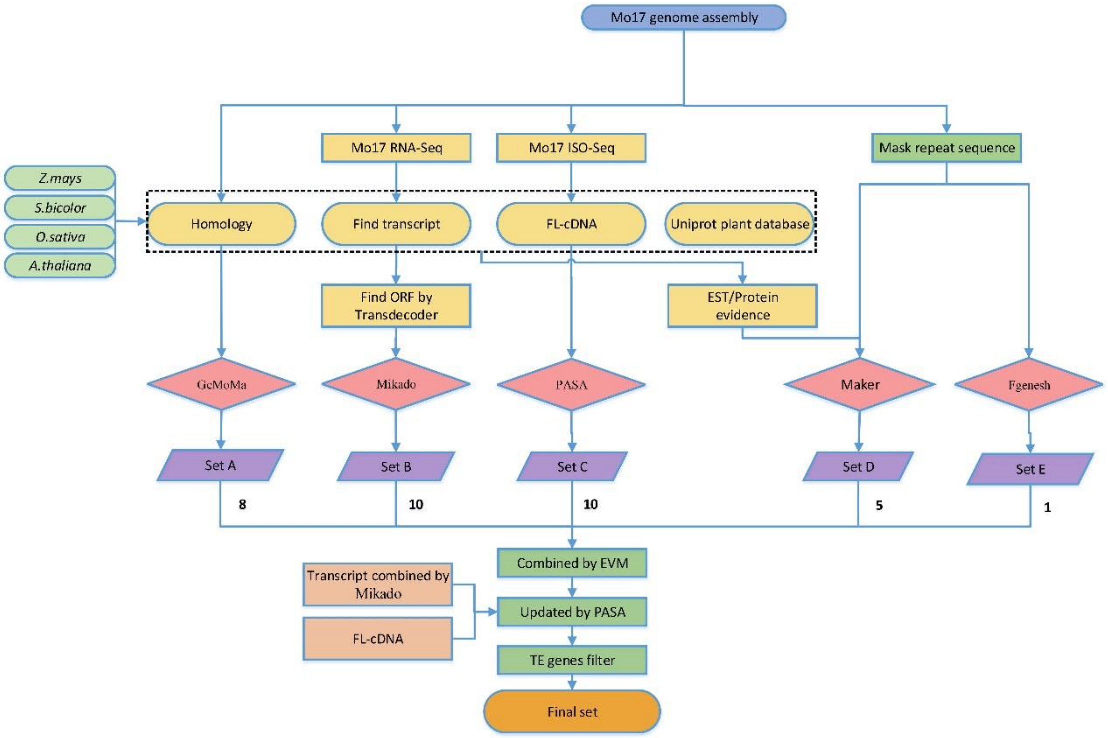

Supplementary Fig. 14. Flowchart showing the method used for annotation of protein coding genes in Mo17 genome.

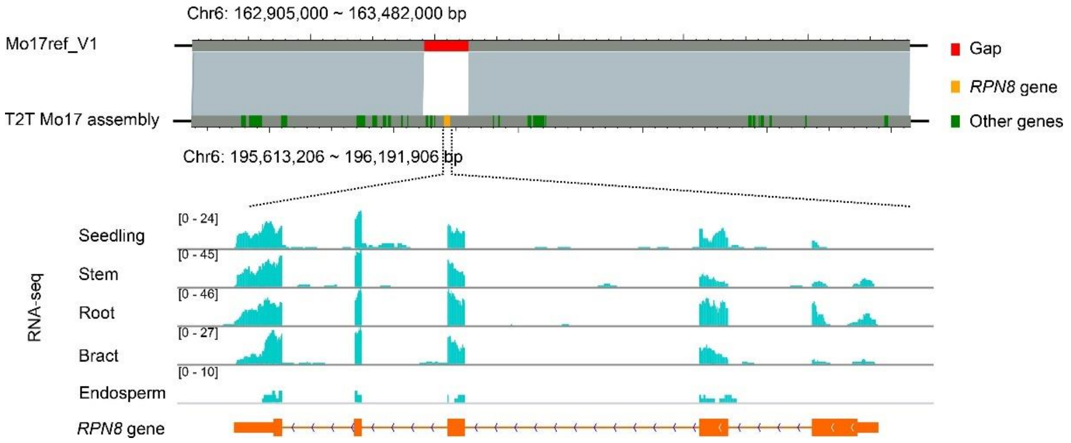

Supplementary Fig. 15. An example of newly assembled gene.

The RNA-seq data of seedling, stem, root, bract and endosperm used here were published previously[4].

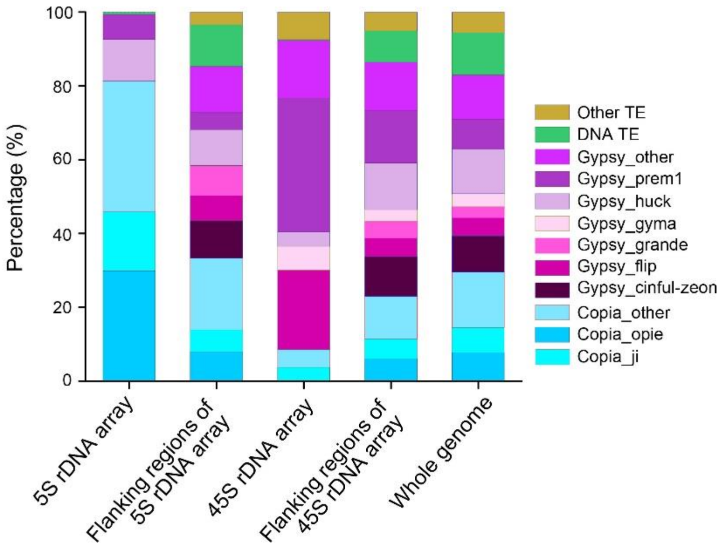

Supplementary Fig. 16. The composition of TEs in rDNA arrays and its flanking regions.

The flanking 500 kb and 43 Mb regions were analyzed for the 5S and 45S rDNA arrays, respectively.

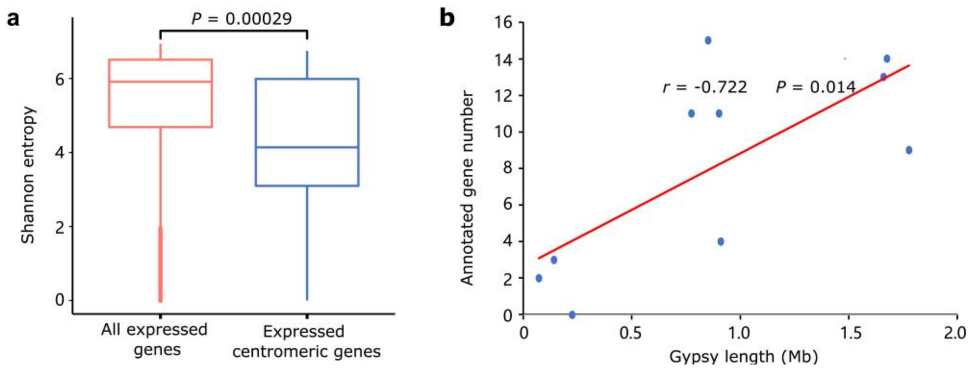

Supplementary Fig. 17. Characteristics of centromeric genes. a) Comparison of the Shannon entropy of all expressed genes (n = 31,826) and expressed centromeric genes (n = 46). The RNA-seq data listed in **Supplementary Table 14** was used for identifying expressed genes (FPKM > 1) and analysis of Shannon entropy. Lower Shannon entropy is observed for centromeric genes suggested that they relatively preferred to be tissue specifically expressed as compared to all expressed genes. In box plots, the 25% and 75% quartiles are shown as lower and upper edges of boxes, respectively, and central lines denote the median. The whiskers extend to 1.5 times the interquartile range. Data beyond the end of the whiskers are displayed as outlying dots. *P*-value was reported from two-tailed t-test without adjustment for multiple comparisons. b) Correlation of annotated gene number on the centromere with the abundant of Gypsy length. For linear correlation, *P* value and coefficient were calculated using Pearson's correlation (two-sided t-test).

Supplementary Tables

Supplementary Table 1. Summary of sequencing data of Mo17 genome.

Supplementary Table 2. Global statistics for the initial Mo17 genome assembly.

Supplementary Table 3. Summary of the assembly of the five TAG repeat arrays related to gaps.

Supplementary Table 4. Statistics of repetitive elements in T2T-assembly of Mo17 genome.

Supplementary Table 5. Statistics of Mo17 and B73 gene models.

Supplementary Table 6. Coordinates and composition of TR-1 arrays in the Mo17 genome.

Supplementary Table 7. Coordinates and composition of knob180 arrays in the Mo17 genome.

Supplementary Table 8. Coordinates and composition of CentC arrays in the Mo17 genome.

Supplementary Table 9. Coordinates and composition of Cent4, tRNAsat, sat112, sat261, and sat268 arrays in the Mo17 genome.

Supplementary Table 10. Coordinates and composition of 5S and 45S rDNA array in the Mo17 genome.

Supplementary Table 11. Coordinates and composition of centromeres defined by CENH3 ChIP-seq in the Mo17 genome.

Supplementary Table 12. Coordinates and composition of telomeres in the Mo17 genome.

Supplementary Table 13. Coordinates and composition of subtelomeres in the Mo17 genome.

Supplementary Table 14. Summary of the RNA-seq data used for gene annotation.

References:

1. Cheng, H., Concepcion, G.T., Feng, X., Zhang, H. & Li, H. Haplotype-resolved de novo assembly using phased assembly graphs with hifiasm. *Nature Methods* **18**, 170-175 (2021).
2. Koren, S. et al. Canu: scalable and accurate long-read assembly via adaptive k-mer weighting and repeat separation. *Genome research* **27**, 722-736 (2017).
3. Page, B.T., Wanous, M.K. & Birchler, J.A. Characterization of a maize chromosome 4 centromeric sequence: evidence for an evolutionary relationship with the B chromosome centromere. *Genetics* **159**, 291-302 (2001).
4. Sun, S. et al. Extensive intraspecific gene order and gene structural variations between Mo17 and other maize genomes. *Nature Genetics* **50**, 1289 (2018).
5. Hufford, M.B. et al. De novo assembly, annotation, and comparative analysis of 26 diverse maize genomes. *Science* **373**, 655-662 (2021).
6. White, R., Pellefigues, C., Ronchese, F., Lamiable, O. & Eccles, D. Investigation of chimeric reads using the MinION. *F1000Res* **6**, 631 (2017).
7. Tan, K., Slevin, M., Meyerson, M. & Li, H. Identifying and correcting repeat-calling errors in nanopore sequencing of telomeres. *Genome Biol* **23**, 180 (2022).
8. Berlin, K. et al. Assembling large genomes with single-molecule sequencing and locality-sensitive hashing. *Nat Biotechnol* **33**, 623-30 (2015).
9. Eid, J. et al. Real-time DNA sequencing from single polymerase molecules. *Science* **323**, 133-138 (2009).
10. Lower, S.S., McGurk, M.P., Clark, A.G. & Barbash, D.A. Satellite DNA evolution: old ideas, new approaches. *Curr Opin Genet Dev* **49**, 70-78 (2018).
11. Volkov, R.A., Komarova, N.Y. & Hemleben, V. Ribosomal DNA in plant hybrids: inheritance, rearrangement, expression. *Systematics and Biodiversity* **5**, 261-276 (2007).
12. McMullen, M.D., Hunter, B., Phillips, R.L. & Rubenstein, I. The structure of the maize ribosomal DNA spacer region. *Nucleic Acids Res* **14**, 4953-68 (1986).
13. Fujisawa, M. et al. Sequence comparison of distal and proximal ribosomal DNA arrays in rice (Oryza sativa L.) chromosome 9S and analysis of their flanking regions. *Theor Appl Genet* **113**, 419-28 (2006).
14. Kurtz, S. et al. Versatile and open software for comparing large genomes. *Genome biology* **5**, R12 (2004).
15. Huang, W. et al. The proteolytic function of the Arabidopsis 26S proteasome is required for specifying leaf adaxial identity. *Plant Cell* **18**, 2479-92 (2006).
16. Naish, M. et al. The genetic and epigenetic landscape of the Arabidopsis centromeres. *Science* **374**, eabi7489 (2021).
17. Li, H. Minimap2: pairwise alignment for nucleotide sequences. *Bioinformatics* **34**, 3094-3100 (2018).
18. Camacho, C. et al. BLAST+: architecture and applications. *BMC bioinformatics* **10**, 421 (2009).
19. Jurka, J. et al. Repbase Update, a database of eukaryotic repetitive elements. *Cytogenetic and genome research* **110**, 462-467 (2005).
20. Li, H. & Durbin, R. Fast and accurate short read alignment with Burrows–Wheeler transform. *Bioinformatics* **25**, 1754-1760 (2009).

21. Marcais, G. & Kingsford, C. A fast, lock-free approach for efficient parallel counting of occurrences of k-mers. *Bioinformatics* **27**, 764-70 (2011).

22. Rhie, A., Walenz, B.P., Koren, S. & Phillippy, A.M. Merqury: reference-free quality, completeness, and phasing assessment for genome assemblies. *Genome Biol* **21**, 245 (2020).

23. Smit, A., Hubley, R. & Green, P. RepeatMasker Open-4.0. 2013–2015. *Institute for Systems Biology. http://repeatmasker. org* (2015).

24. Ou, S. et al. Benchmarking transposable element annotation methods for creation of a streamlined, comprehensive pipeline. *Genome Biol* **20**, 275 (2019).

25. Solovyev, V., Kosarev, P., Seledsov, I. & Vorobyev, D. Automatic annotation of eukaryotic genes, pseudogenes and promoters. *Genome biology* **7**, S10 (2006).

26. Chen, S., Zhou, Y., Chen, Y. & Gu, J. fastp: an ultra-fast all-in-one FASTQ preprocessor. *Bioinformatics* **34**, i884-i890 (2018).

27. Dobin, A. et al. STAR: ultrafast universal RNA-seq aligner. *Bioinformatics* **29**, 15-21 (2013).

28. Pertea, M. et al. StringTie enables improved reconstruction of a transcriptome from RNA-seq reads. *Nature biotechnology* **33**, 290-295 (2015).

29. Trapnell, C. et al. Differential gene and transcript expression analysis of RNA-seq experiments with TopHat and Cufflinks. *Nature protocols* **7**, 562-578 (2012).

30. Song, L., Sabunciyan, S. & Florea, L. CLASS2: accurate and efficient splice variant annotation from RNA-seq reads. *Nucleic Acids Res* **44**, e98 (2016).

31. Niknafs, Y.S., Pandian, B., Iyer, H.K., Chinnaiyan, A.M. & Iyer, M.K. TACO produces robust multisample transcriptome assemblies from RNA-seq. *Nat Methods* **14**, 68-70 (2017).

32. Venturini, L., Caim, S., Kaithakottil, G.G., Mapleson, D.L. & Swarbreck, D. Leveraging multiple transcriptome assembly methods for improved gene structure annotation. *Gigascience* **7**, giy093 (2018).

33. Mapleson, D., Venturini, L., Kaithakottil, G. & Swarbreck, D. Efficient and accurate detection of splice junctions from RNA-seq with Portcullis. *Gigascience* **7**, giy131 (2018).

34. Li, H. et al. The sequence alignment/map format and SAMtools. *Bioinformatics* **25**, 2078-2079 (2009).

35. Haas, B.J. et al. De novo transcript sequence reconstruction from RNA-seq using the Trinity platform for reference generation and analysis. *Nat Protoc* **8**, 1494-512 (2013).

36. Buchfink, B., Xie, C. & Huson, D.H. Fast and sensitive protein alignment using DIAMOND. *Nat Methods* **12**, 59-60 (2015).

37. Haas, B.J. et al. Improving the Arabidopsis genome annotation using maximal transcript alignment assemblies. *Nucleic Acids Res* **31**, 5654-66 (2003).

38. Wu, T.D. & Watanabe, C.K. GMAP: a genomic mapping and alignment program for mRNA and EST sequences. *Bioinformatics* **21**, 1859-75 (2005).

39. Steinegger, M. & Soding, J. MMseqs2 enables sensitive protein sequence searching for the analysis of massive data sets. *Nat Biotechnol* **35**, 1026-1028 (2017).

40. Grau, J. et al. Jstacs: a Java framework for statistical analysis and classification of biological sequences. *The Journal of Machine Learning Research* **13**, 1967-1971 (2012).

41. Campbell, M.S., Holt, C., Moore, B. & Yandell, M. Genome Annotation and Curation Using MAKER and MAKER-P. *Curr Protoc Bioinformatics* **48**, 4.11.1-39 (2014).

42. Haas, B.J. et al. Automated eukaryotic gene structure annotation using EVidenceModeler and the Program to Assemble Spliced Alignments. *Genome biology* **9**, 1-22 (2008).

43. Blum, M. et al. The InterPro protein families and domains database: 20 years on. *Nucleic Acids Res* **49**, D344-D354 (2021).
