# Supplementary material for: A complete telomere-to-telomere assembly of the maize genome
Source: Nat Genet. 2023 Jun 15;55(7):1221–31. doi: 10.1038/s41588-023-01419-6 (PMC10335936; doi:10.1038/s41588-023-01419-6)
Supplement: Supplementary file 2 — Reporting Summary [file 41588_2023_1419_MOESM2_ESM.pdf]

nature portfolio

Corresponding author(s): Jinsheng Lai

Last updated by author(s): 2023-4-17

# Reporting Summary

Nature Portfolio wishes to improve the reproducibility of the work that we publish. This form provides structure for consistency and transparency in reporting. For further information on Nature Portfolio policies, see our Editorial Policies and the Editorial Policy Checklist.

## Statistics

For all statistical analyses, confirm that the following items are present in the figure legend, table legend, main text, or Methods section.

| n/a | Confirmed | |
|---|---|---|
| ☐ | ☒ | The exact sample size (*n*) for each experimental group/condition, given as a discrete number and unit of measurement |
| ☐ | ☒ | A statement on whether measurements were taken from distinct samples or whether the same sample was measured repeatedly |
| ☐ | ☒ | The statistical test(s) used AND whether they are one- or two-sided<br>*Only common tests should be described solely by name; describe more complex techniques in the Methods section.* |
| ☒ | ☐ | A description of all covariates tested |
| ☐ | ☒ | A description of any assumptions or corrections, such as tests of normality and adjustment for multiple comparisons |
| ☐ | ☒ | A full description of the statistical parameters including central tendency (e.g. means) or other basic estimates (e.g. regression coefficient) AND variation (e.g. standard deviation) or associated estimates of uncertainty (e.g. confidence intervals) |
| ☐ | ☒ | For null hypothesis testing, the test statistic (e.g. *F*, *t*, *r*) with confidence intervals, effect sizes, degrees of freedom and *P* value noted<br>*Give P values as exact values whenever suitable.* |
| ☒ | ☐ | For Bayesian analysis, information on the choice of priors and Markov chain Monte Carlo settings |
| ☒ | ☐ | For hierarchical and complex designs, identification of the appropriate level for tests and full reporting of outcomes |
| ☐ | ☒ | Estimates of effect sizes (e.g. Cohen's *d*, Pearson's *r*), indicating how they were calculated |

*Our web collection on statistics for biologists contains articles on many of the points above.*

## Software and code

Policy information about availability of computer code

| | |
|---|---|
| Data collection | PacBio SequelI platform produced HiFi SMRT long reads. Nanopore PromethION platform produced ONT common and ultra-long reads. PacBio Sequel platform produced ISO-seq reads. Illumina HiSeq platform produced ChIP-seq reads. |
| Data analysis | Genome assembly: NextDenovo v2.2-beta.0, NextPolish v1.1.0, Hifiasm v.0.7, Canu v.2.0; Gap closure: Minimap2 v2.17, BLASTN v2.9.0, Solve v3.5.1; Satellite analysis: MAFFT v7.475; Genome evaluation: Merqury v1.1, BWA v0.7.17; Genome annotation: EDTA v1.7.0, RepeatMasker v4.1.1, trf v4.09 , Fgenesh v7.2.2, STAR v2.7.8a, FASTP v0.20.0, StringTie v2.1.2, Cufflinks v2.2.1, CLASS2 v2.1.7, TACO v0.7.3, Mikado v2.0rc2, SAMTools v1.9, TransDecoder v5.5.0, Diamond v2.0.1, PASA v2.3.3, GMAP v.2017-11-15, MMseqs v12.113e3, GeMoMa v1.6.4, MAKER v 2.31.10, InterProScan v5.39-77.0; Centromere identification: Bowtie2 v2.4.4, Deeptools v3.5.1, BEDTools v2.29.2; Identification of duplicated genes: McscanX(https://github.com/wyp1125/MCScanX), OrthoFinder v2.5.2; Customized scripts in GitHub repository: https://github.com/LAILAB-CAU/update-Mo17. |

For manuscripts utilizing custom algorithms or software that are central to the research but not yet described in published literature, software must be made available to editors and reviewers. We strongly encourage code deposition in a community repository (e.g. GitHub). See the Nature Portfolio guidelines for submitting code & software for further information.

## Data

Policy information about availability of data

All manuscripts must include a data availability statement. This statement should provide the following information, where applicable:

- Accession codes, unique identifiers, or web links for publicly available datasets
- A description of any restrictions on data availability
- For clinical datasets or third party data, please ensure that the statement adheres to our policy

The genome assembly and raw sequencing data generated in this study, including PacBio HiFi data, common ONT data, ultra-long ONT data, ISO-seq data, and ChIP-seq data can be achieved from NCBI with BioProject number PRJNA751841. The GenBank accession number of the above data is JAIRCI000000000. The .fast5 format files of the ultra-long ONT reads have been deposited in the National Genomics Data Center (NGDC), Beijing Institute of Genomics, Chinese Academy of Sciences, under BioProject accession number PRJCA012690. Genome assembly and gene annotation files can also be found in CyVerse(https://data.cyverse.org/dav-anon/iplant/home/laijs/Zm-Mo17-REFERENCE-CAU-2.0/). The Illumina PCR-free data used in this study can be obtained from NCBI under accession number SRP111315. The RNA-seq data used for gene annotation can be achieved from NCBI under accession numbers of GSE16916, GSE54272, GSE57337, GSE61810, GSE70192, GSE43142, SRP051572, SRP064910, SRP052226, SRP006703, SRP009313, SRP010124, SRP011187, SRP011480, SRP013432, SRP015339, SRP110782, SRP111315, SRP017111, SRP018088, SRP026161, and SRP029742. The detail runs of published RNA-seq data used are demonstrated in Supplementary Table 14.

# Field-specific reporting

Please select the one below that is the best fit for your research. If you are not sure, read the appropriate sections before making your selection.

☒ Life sciences ☐ Behavioural & social sciences ☐ Ecological, evolutionary & environmental sciences

For a reference copy of the document with all sections, see nature.com/documents/nr-reporting-summary-flat.pdf

# Life sciences study design

All studies must disclose on these points even when the disclosure is negative.

| | |
|---|---|
| Sample size | The sample size for genome assembly was the number of inbred lines. We choose only one maize inbred lines (Mo17) for sequencing and assembly.<br>The sample size for the statistic analysis of each experiment was clearly mentioned in each figure legend or Methods. |
| Data exclusions | Only quality filtered ultra-long ONT reads were used for ONT data based assembly. |
| Replication | Replications for each experiment were clearly stated in figure legends or Methods section.<br>Replications of repeated experiments were evaluated by proper statistic analyses and confirmed to be successful. |
| Randomization | For each individual of Mo17 inbred line, the sampling process for DNA sequencing, ISO-seq and ChIP-seq was randomly conducted. |
| Blinding | Blinding is not necessary for genome sequencing and assembly, since the investigators know which maize species they were handling. No blinding should not affect interpretation as all our experiment measures were objective. |

# Reporting for specific materials, systems and methods

We require information from authors about some types of materials, experimental systems and methods used in many studies. Here, indicate whether each material, system or method listed is relevant to your study. If you are not sure if a list item applies to your research, read the appropriate section before selecting a response.

### Materials & experimental systems

| n/a | Involved in the study | |
|---|---|---|
| ☐ | ☒ | Antibodies |
| ☒ | ☐ | Eukaryotic cell lines |
| ☒ | ☐ | Palaeontology and archaeology |
| ☒ | ☐ | Animals and other organisms |
| ☒ | ☐ | Human research participants |
| ☒ | ☐ | Clinical data |
| ☒ | ☐ | Dual use research of concern |

### Methods

| n/a | Involved in the study | |
|---|---|---|
| ☐ | ☒ | ChIP-seq |
| ☒ | ☐ | Flow cytometry |
| ☒ | ☐ | MRI-based neuroimaging |

## Antibodies

| | |
|---|---|
| Antibodies used | CENH3 antibody. The CENH3 antibody is a rabbit polyclonal against the peptide RPGTVALREIRKYQKSSTSATPERAAGTGGR. The |

| | |
|---|---|
| Antibodies used | antibodies were custom-produced and supplied by GL Biochem. |
| Validation | The rabbit polyclonal antibody against CENH3 was validated to be available according to a previous report (Fu, Shulan, et al. "De novo centromere formation on a chromosome fragment in maize." Proceedings of the National Academy of Sciences 110.15 (2013): 6033-6036), in which the anti-CENH3 antibody used is same as we used here. |

# ChIP-seq

## Data deposition

☒ Confirm that both raw and final processed data have been deposited in a public database such as GEO.

☒ Confirm that you have deposited or provided access to graph files (e.g. BED files) for the called peaks.

| | |
|---|---|
| Data access links<br>*May remain private before publication.* | https://www.ncbi.nlm.nih.gov/bioproject/?term=PRJNA751841 |
| Files in database submission | SRR21509776, SRR21509777, SRR21509778, SRR21509779 |
| Genome browser session<br>(e.g. UCSC) | no longer applicable |

## Methodology

| | |
|---|---|
| Replicates | Two biological replicates were set |
| Sequencing depth | Depth 10 X; total reads, 158,687,990; mapped reads 58,605,672, unique mapped reads 27,806,396; 150 bp pared-end reads |
| Antibodies | anti-CENH3 |
| Peak calling parameters | Enrichment level of CENH3 for each base was obtained using bamCompare in the Deeptools packag (v3.5.1) with the parameters of '--binSize 1 --numberOfProcessors 40 --operation ratio --outFileFormat bedgraph'. Average enrichment of each 1 kb-bin of the genome was then calculated. The bins that enrichment levels greater than 5 were retained, which with a distance interval less than 1 Mb were merged. The final centromeric regions were determined by visual inspection of the distribution of CENH3 ChIP-seq peaks. |
| Data quality | All ten centromeres in maize were successfully identified. |
| Software | Enrichment level of CENH3 for each base was obtained using bamCompare in the Deeptools package (v3.5.1). |
